# Gene regulation network inference using k-nearest neighbor-based mutual information estimation: revisiting an old DREAM

**DOI:** 10.1186/s12859-022-05047-5

**Published:** 2023-03-06

**Authors:** Lior I. Shachaf, Elijah Roberts, Patrick Cahan, Jie Xiao

**Affiliations:** 1 Department of Biophysics, Johns Hopkins University, 3400 N. Charles Street, Baltimore, MD 21218 USA; 2 Present Address: 10x Genomics, 6230 Stoneridge Mall Road, Pleasanton, CA 94588-3260 USA; 3 Department of Biomedical Engineering, Department of Molecular Biology and Genetics, Institute for Cell Engineering, Johns Hopkins School of Medicine, 733 N. Broadway, Baltimore, MD 21205 USA; 4 Department of Biophysics and Biophysical Chemistry, Johns Hopkins School of Medicine, 725 N. Wolfe Street, WBSB 708, Baltimore, MD 21205 USA

**Keywords:** Gene regulatory network inference, Mutual information, k-nearest neighbor

## Abstract

**Background:**

A cell exhibits a variety of responses to internal and external cues. These responses are possible, in part, due to the presence of an elaborate gene regulatory network (GRN) in every single cell. In the past 20 years, many groups worked on reconstructing the topological structure of GRNs from large-scale gene expression data using a variety of inference algorithms. Insights gained about participating players in GRNs may ultimately lead to therapeutic benefits. Mutual information (MI) is a widely used metric within this inference/reconstruction pipeline as it can detect any correlation (linear and non-linear) between any number of variables (*n*-dimensions). However, the use of MI with continuous data (for example, normalized fluorescence intensity measurement of gene expression levels) is sensitive to data size, correlation strength and underlying distributions, and often requires laborious and, at times, ad hoc optimization.

**Results:**

In this work, we first show that estimating MI of a bi- and tri-variate Gaussian distribution using *k*-nearest neighbor (kNN) MI estimation results in significant error reduction as compared to commonly used methods based on fixed binning. Second, we demonstrate that implementing the MI-based kNN Kraskov–Stoögbauer–Grassberger (KSG) algorithm leads to a significant improvement in GRN reconstruction for popular inference algorithms, such as Context Likelihood of Relatedness (CLR). Finally, through extensive in-silico benchmarking we show that a new inference algorithm CMIA (Conditional Mutual Information Augmentation), inspired by CLR, in combination with the KSG-MI estimator, outperforms commonly used methods.

**Conclusions:**

Using three canonical datasets containing 15 synthetic networks, the newly developed method for GRN reconstruction—which combines CMIA, and the KSG-MI estimator—achieves an improvement of 20–35% in precision-recall measures over the current gold standard in the field. This new method will enable researchers to discover new gene interactions or better choose gene candidates for experimental validations.

**Supplementary Information:**

The online version contains supplementary material available at 10.1186/s12859-022-05047-5.

## Background

Most cells in a multicellular organism contain the same genome, yet they can differentiate into different cell types and adapt to different environmental conditions [1]. These responses to internal and external cues are possible due to the presence of an elaborate gene regulatory network (GRN). A GRN is the genome’s “flowchart“ for various biological processes such as sensing, development, and metabolism, enabling the cell to follow specific instructions upon an internal or external stimulation. Understanding how genomic flowcharts are organized brings the potential to remediate dysfunctional ones [2] and design new ones for synthetic biology [3].

Advances in large-scale gene expression data collected from omic-level microarrays and RNA-seq experiments allow the construction of basic networks by clustering co-expressed genes using statistical correlation metrics such as covariance and threshold to determine the statistical significance [4]. Another common practice is to monitor the expression of multiple genes in response to perturbations and then infer the relationship between these genes [5]. Currently, there are several classes of methods to infer GRNs from expression data, such as the Bayesian networks method, the statistical/information theory method, and ordinary differential equations (ODEs) (see excellent reviews [6–8]).

Originally introduced for communication systems by Shannon in the late 40s [9], mutual information (MI) was quickly adopted by other disciplines as a statistical tool to evaluate the dependence between variables. Unlike the abovementioned traditional correlation methods like covariance, MI can detect linear and non-linear relationship between variables and can be applied to test the dependence between any number of variables (*n*-dimensions).

Over the last 20 years, researchers have implemented many methods employing MI to reconstruct GRNs, such as Relevance Networks [10]; ARACNE (Algorithm for the Reconstruction of Accurate Cellular Networks [11]); and CLR (Context Likelihood of Relatedness [12]). Using MI with two variables (i.e. genes) is straightforward, but due to the positive and symmetric nature of two-way MI [13], MI with only two variables cannot distinguish between direct and indirect regulation, coregulation, or logical gate-type interactions [14, 15]. To overcome these issues, a few groups have used different three-dimensional MI measures in inference algorithms [14, 16, 17] (for a comprehensive list of methods, see Mousavian et al. [18]). Importantly, in most methods using MI, continuous input (*i.e.*, normalized fluorescence intensity data for gene expression) needs to be discretized first to build probability density functions (PDF). This practice is known to be sensitive to data size, correlation strength and underlying distributions [19].

**Fig. 1:**
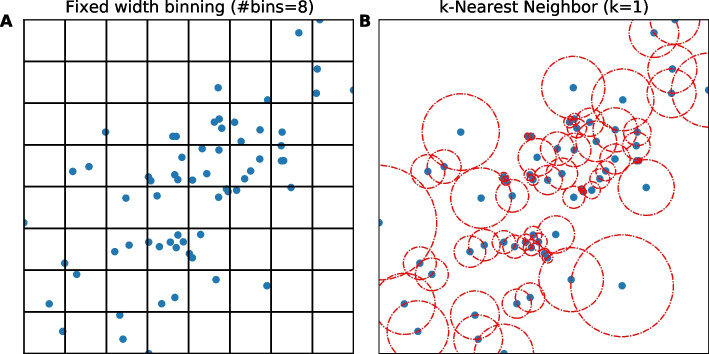
Illustration of two methods to evaluate distribution: **A** Fixed width binning, and **B** k-Nearest-Neighbor (k = 1). Data points are shown as blue circles, bin edges are shown in black, and distances to k = 1 neighbor as the radius of dashed red circles

In general, the simplest and most computationally inexpensive method to discretize continuous data is fixed (width) binning (FB) (Fig. 1A), where a histogram with a fixed number of bins (or bin width) determined by certain statistical rules is used to model the PDF. For finite data size, FB generally under- or over-estimates MI (Additional file 2: Fig. S1A). Over the years, researchers developed different methods to mitigate bin number sensitivity and to better estimate (or correct the bias in) MI, especially for data of small sizes. These methods correct either the entropies (Miller–Madow [20]) or the probability distribution by adaptive partitioning (AP) [21], k-Nearest Neighbor (kNN) [22] (Fig. 1B), kernel density estimator (KDE) [14] and/or B-spline functions, in which data points are divided into fractions between a predefined number of adjacent bins [23]. Unfortunately, all these methods make assumptions on the density distribution and require adjustment of parameters by the user for different scenarios except for kNN, which is shown to be accurate and robust across different values of *k* [19, 22]. However, kNN is rarely used due to the higher computational costs it entailed [24] or the limited improvement for two variables (2d) in downstream analysis.

The problem of accurately estimating the correlation between genes has only worsened in this new era of single cell transcriptome studies, as data is larger yet sparser, often with non-Gaussian distributions. In this work, we focus on two subjects: (a) Improving MI estimation—we present an implementation of a three-way MI estimator based on kNN, which addresses large errors in estimating MI measures for three variables (3d). (b) Improving GRN inference—we present CMIA (Conditional Mutual Information Augmentation), a novel inference algorithm inspired by Synergy-Augmented CLR (SA-CLR) [17]. By testing various mutual information estimators against the ground truth solved from an analytical solution and comparing their performance using in-silico GRN benchmarking data, we find that kNN-based three-way MI estimator Kraskov-Stoögbauer-Grassberger (KSG) improves the performance of common GRN inference methods. Together with the inference algorithm, CMIA, it outperforms other commonly used GRN reconstruction methods in the field.

## Results

### Benchmark MI estimations of a Gaussian distribution

To evaluate the performance of different mutual information (MI) estimators on continuous data, we calculated their deviations from the true underlying value by defining a percent error:

$$percent \: error = \frac{{\left| {MI_{Analytical} - MI_{Estimated}} \right|}}{MI_{Analytical}} \times 100\%$$

**Fig. 2:**
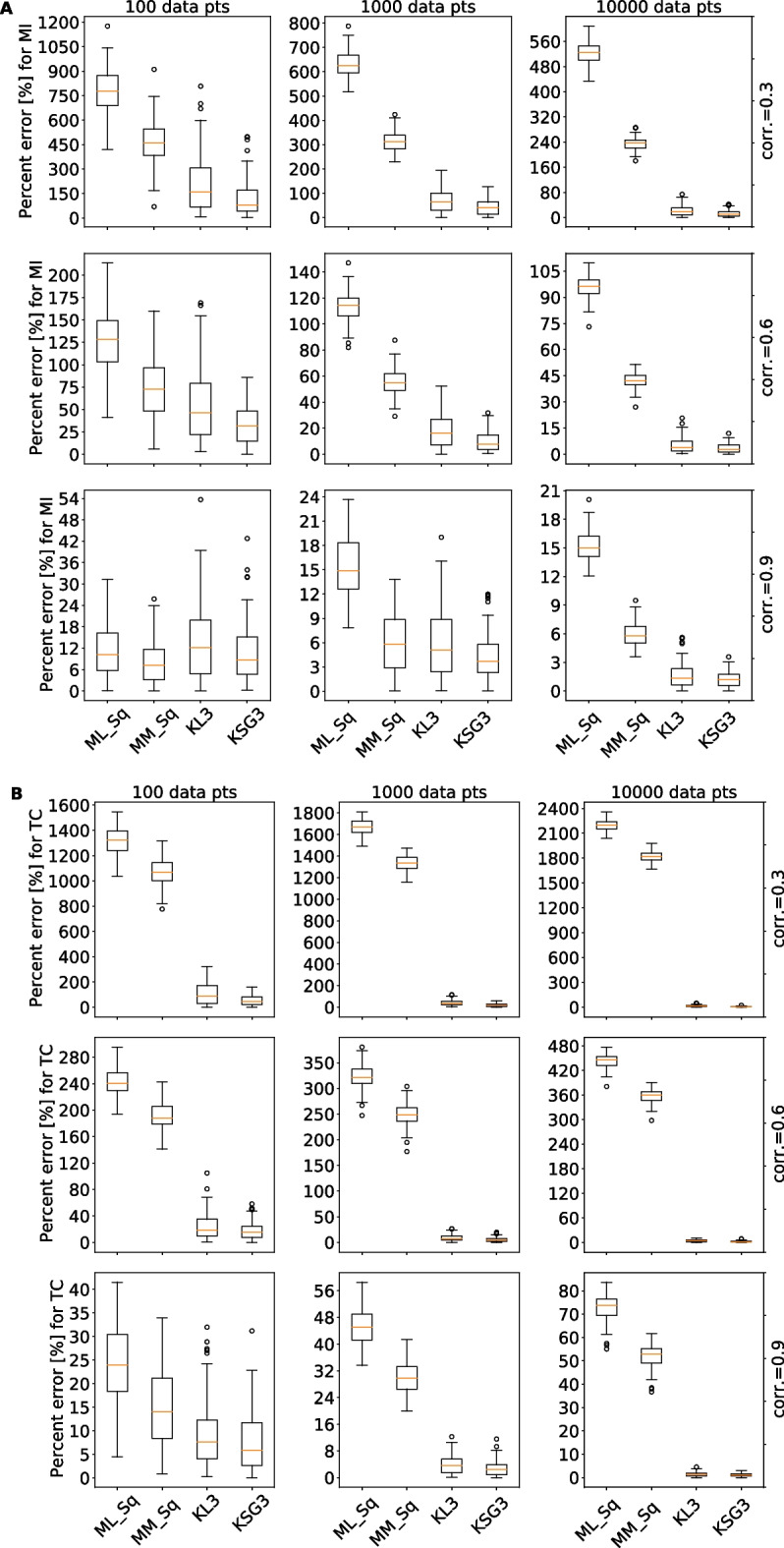
Percent error of different mutual information estimators for multivariate gaussian distribution. Each boxplot represents 100 replicates, with columns representing sample size = {100,1K,10K}, and rows the correlation = {0.3,0.6,0.9}. **A** Percent error (y-axis) for two-way mutual information (MI2) was compared for 4 different methods: ML_Sq = Maximum Likelihood (Shannon’s MI) with fixed width binning (number of bins is determined by square-root), MM_Sq = Miller–Madow’s formula for MI with square-root for the number of bins, KL3 = KL formula for kNN-MI with k = 3, KSG3 = KSG formula for kNN-MI with k = 3; **B** same methods compared for total correlation (TC)

In most biologically relevant cases, one does not know what the true MI value is, because one does not know the probability distributions of the variables we are concerned with. Nevertheless, the true underlying value of MI of a few distributions such as Gaussian distribution can be analytically calculated. Therefore, to allow quantitative comparisons between different MI estimators, we used the analytical solution of Shannon’s entropy for a Gaussian distribution (see Additional file 1: Appendix S2) to calculate the MI by entropy summation (Table 1). We then compared all methods of different data sizes (100, 1K, 10K, referring to the number of different conditions/perturbations/time points of individual genes) and different correlation strengths (0.3, 0.6, 0.9) between two or three variables (number of genes, 2d or 3d) drawn from a Gaussian distribution with a mean at zero and a variance of one (the absolute values of mean and variance are not important in the calculation as the final solution only contains correlation, see Additional file 1: Appendix S2). For two-way MI (two variables, or 2d), we compared the following MI estimators: (i) Maximum Likelihood (ML, given by Shannon, Table 1), (ii) Miller–Madow correction (MM, see Additional file 1: Appendix S3), (iii) Kozachenko–Leonenko (KL) [25], and (iv) KSG (Fig. 2A). The first two methods use FB to discretize the continuous data, and in general the best number of bins changes depending on the data size and correlation between variables (Additional file 2: Fig. S1A). As a priori the correlation strength is unknown, for the number of bins we used the common practice $\sqrt N$, where N equals the number of data points, and the result was rounded down to align with methods in the next section. The latter two methods both use kNN, and we found that any selection of *k* resulted in good alignment with the analytical solution (see Additional file 2: Figure S1B). We chose the third nearest-neighbor (*k* = 3) as recommended by Kraskov et al. [22] because a *k* value of 3 resulted in a good trade-off between precision and computational cost. As shown in Fig. 2, in all cases the two kNN-based MI estimators performed well similarly and outperformed the fixed-binning methods judged by the percentage error.

**Table 1:** Mutual Information formalism

Term	Symbol	Formula	Venn diagrams
Shannon’s entropy of X	H(X)	$- \mathop \sum \limits_{x} p\left( x \right)\log p\left( x \right)$	
Joint entropy of X & Y	H(X,Y)	$- \mathop \sum \limits_{x} \mathop \sum \limits_{y} p\left( {x,y} \right)\log p\left( {x,y} \right)$	
Joint entropy of X,Y & Z	H(X,Y,Z)	$- \mathop \sum \limits_{x} \mathop \sum \limits_{y} \mathop \sum \limits_{z} p\left( {x,y,z} \right)\log p\left( {x,y,z} \right)$	
Two-way Mutual Information	MI(X;Y)	H(X) + H(Y) − H(X,Y)	
Total Correlation	TC(X,Y,Z)	H(X) + H(Y) + H(Z) − H(X,Y,Z)	
Three-way MI	MI3((X,Y);Z)	TC − MI(X;Y)	
Interaction Information	II(X,Y,Z)	TC − MI(X;Y) − MI(X;Z) − MI(Y;Z)	
Conditional MI	CMI(X;Y|Z)	TC − MI(X;Z) − MI(Y;Z)

While two-way MI estimators were studied extensively [22, 26], to our knowledge, no benchmark was done on MI with three or more variables. We repeated the same methodology described above but this time for the 3d Total Correlation (TC) (Fig. 2B, Additional file 2: Figure S1C–D). Similar to the 2d case, kNN-based MI estimators KL3 and KSG3 outperformed the other methods. We also examined the other three-way MI quantities, three-way MI (MI3), Interaction Information (II), Conditional Mutual Information (CMI) (see Additional file 2: Figure S2–4) and obtained similar results. We also explored whether a higher kNN value, for example k = 10, further improved accuracy. We found that a higher k value (k = 10) does not improve the accuracy dramatically compared to that in k = 3 (Additional file 2: Figure S5–6), but it did reduce the variance for small correlations (r = 0.3).

### In-silico GRN Inference performance enhancement

Next, we aim to investigate whether the high precision of MI estimation based on kNN for bi- and tri-variate Gaussian distributions also translates to a higher performance in inferring GRN structure compared to other MI estimation methods described above.

To compare the performance of different MI estimators and inference algorithms, we used a total of 15 different synthetic networks: ten synthetic networks from the DREAM3 (Dialogue for Reverse Engineering Assessments and Methods) competition [27] with 50 and 100 genes, respectively, and five networks from DREAM4 with 100 genes. The networks were extracted from documented regulation databases of *Escherichia* *coli* and *Saccharomyces* *cerevisiae* [28]. We used the software GeneNetWeaver 3.1.2b [29] with default settings to generate simulated expression data for each network and performed ten replicates to include the variance in expression data due to stochastic molecular noise. Furthermore, to comply with the majority of available experimental data, we only used the simulated steady-state data (Wild type, knockouts, dual-knockouts, knockdowns, multifactorial perturbation) accumulating to 170, 169 and 201 conditions in the 50 gene synthetic networks for *E.* *coli* 1, *E.* *coli* 2 and Yeast1/2/3 respectively, 341, 322 and 401 conditions in the DREAM3 100 gene synthetic networks for *E.* *coli* 1, *E.* *coli* 2 and Yeast1/2/3 respectively, and 393, 401 conditions in the DREAM4 100 gene networks. We then ran the expression data through our custom Python 3.8 code pipeline to calculate the area under precision–recall curve (AUPR) for each replicate.

**Fig. 3:**
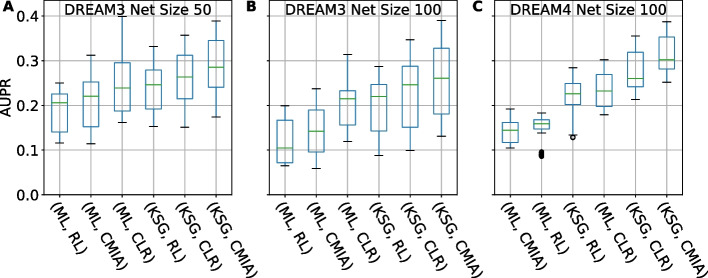
AUPR values for different combinations of MI estimator (ML or KSG) and GRN inference algorithm (RL, CLR or CMIA). **A** Sorted boxplots showing networks of size 50 from DREAM3, **B** Networks of size 100 from DREAM3, **C** Networks of size 100 from DREAM4. For the different network sizes each boxplot represents 50 networks (5 different networks × 10 replicates). Green bar inside each boxplot represents median AUPR value

In Fig. 3 we show sorted boxplots of the AUPR values (*y*-axis) comparing six combinations of three inference algorithms (Relevance Networks, RL; Context-Likelihood-Relatedness, CLR; and our Conditional-Mutual-Information-Augmentation, CMIA) and two MI estimators (ML, fixed bin-based; KSG, kNN-based), for five networks with 50 genes (Fig. 3A), five networks of 100 genes from DREAM3 (Fig. 3B), and five networks of 100 genes from DREAM4 (Fig. 3C). In all cases, the kNN-based KSG as the MI estimator improves the performance of the inference algorithms. The improvement is more significant for CMIA, which uses three-way MI calculations, and corroborate the higher percent error we found when estimating TC (Fig. 2B). It is important to note that using AUPR alone can miss significant differences in the precision and recall results between methods. To complement this comparison, we computed the precision–recall curves of one representative replicate (rep_2) using the six methods for the five different *in-silico* networks of size 100 from DREAM4 (Additional file 2: Fig. S7). We observed that replacing the MI estimator from ML to KSG improved the precision-recall, and our method of choice {KSG,CMIA} outperformed the gold standard {ML,CLR}.

To further decouple the effect on performance between the MI estimators (KSG vs. ML) and the inference algorithms (CMIA vs. CLR), we scanned different *k* values in the range {1–15} and compared that with bin-scanning in the range {2–20}. For both the inference algorithms CLR and CMIA (Additional file 2: Figure S8), a *k* value of greater or equal to 2 has the least variations on the resulting AUPR, whereas different bin numbers give variable AUPR values. Although some specific bin numbers used in the ML estimator (for example, between 3 and 10 in ML-CLR) give higher AUPR values than the corresponding kNN estimator KSG, the stability of the kNN estimator is advantageous when the best bin number cannot be determined.

To confirm that our computational pipeline and choice of MI estimators and inference algorithms perform better than a random baseline, for the five synthetic networks (each with 10 replicates) of size 50 genes from DREAM3, we randomly permuted 100 times each expression row (representing the expression value of 50 genes under a specific condition/perturbation). We then calculated and plotted the corresponding AUPR for the six pairs of combinations. We observed that the randomized dataset resulted in more than two-fold lower AUPR for all six combinations (Additional file 2: Figure S9A) compared to that of non-randomized dataset (Additional file 2: Figure S9B), demonstrating that the improvement we observed was specific for the networks investigated.

### In-silico GRN Inference performance comparison

**Fig. 4:**
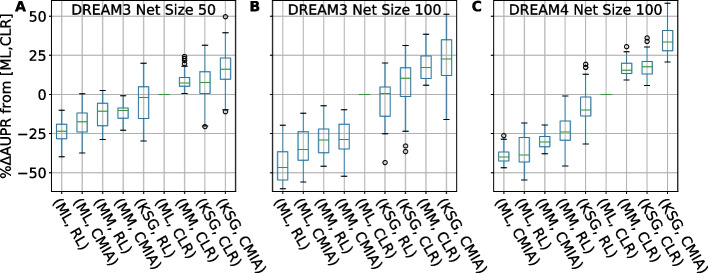
AUPR difference of combinations of MI estimators and inference algorithms relative to the gold standard {ML,CLR}. **A** Sorted boxplots showing comparison for network size of 50 from DREAM3, **B** and size of 100 from DREAM3, **C** size of 100 from DREAM4. Each boxplot represents 50 networks (5 different networks X 10 replicates). Green bar inside each boxplot represents median AUPR value. A complete list of tested GRN inference algo & MI estimators can be found in Additional file 2: Table S1

To verify whether the performance enhancement introduced by kNN-based MI estimators is general for other GRN inference algorithms, we further extended our benchmark to twenty-four different combinations of the four MI estimators (discrete bin-based ML and MM, and kNN-based KL, and KSG) with six inference algorithms described in the Methods section (RL, CLR, ARACNE, SA-CLR, CMIA, CMI2rt) and compared them to the field gold standard combination {ML, CLR} (Fig. 4). To compare the performance differences quantitatively, we calculated the change in AUPR for each replicate relative to the field’s gold standard combination of CLR inference algorithm with ML for MI calculations. In Fig. 4 we show the top nine combinations, omitting ARACNE and CMI2rt among the inference algorithms, and KL from the MI estimators because of their poor performance. We also omitted SA-CLR due to its similarity to CLR and CMIA (see full data in Additional file 3: Table S1). The combination of {KSG,CMIA} gave the best median score in the combined networks inspected under each category. It showed a median improvement of 16% and 24% for networks of 50 and 100 genes from DREAM3, respectively (Fig. 4A, B), and 34% improvements for networks of 100 genes from DREAM4 (Fig. 4C). Furthermore, replacing the MI estimator from ML to KSG in the case of the gold standard (ML,CLR} can lead to significant improvement in GRN reconstruction performance, with median increase in AUPR of 8–18%.

Inference algorithms based on MI are only a subset of available methods to reconstruct GRN as described in Pratapa et al*.* [30] and Bansal et al*.* [6]. In this work we chose to focus on improving MI based inference algorithms. Nevertheless, using the BEELINE benchmark [30]*,* we have access to 12 different inference algorithms and chose to add the two recommended GRN inference algorithms to our comparison: PIDC (with maximum likelihood MI estimator) and GRNBOOST2 (based on gradient-boosted-trees, a supervised machine learning technique) for their accuracy and consistent performances. Both PIDC and GRNBOOST2 achieved lower AUPR values compared to the gold standard of {ML,CLR}, which we used as a comparison baseline in our work (Additional file 2: Figure S10).

### In-silico GRN Inference performance of different organisms

**Fig. 5:**
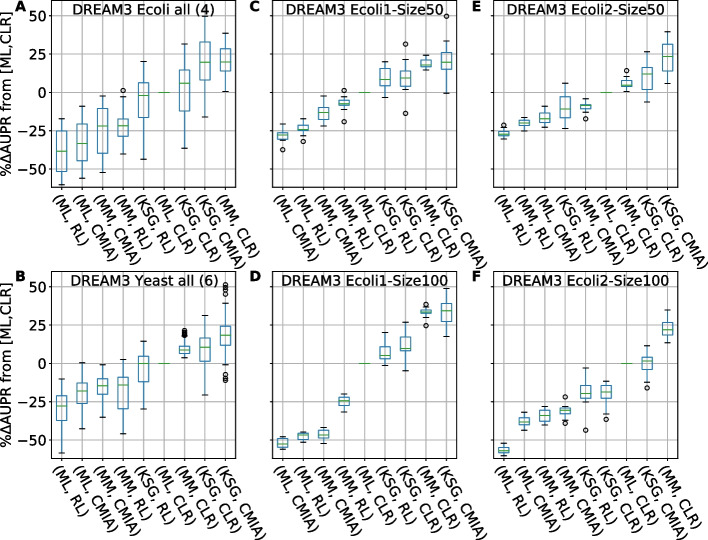
Performance comparison of GRN reconstruction for different in-silico networks modeled from *E. coli* & Yeast. x-axis shows different combinations of [MI estimator, inference algo], y-axis shows percentage AUPR difference (increase or decrease) relative to the gold standard combination [ML,CLR]. **A** Sorted boxplots of the combined four *E. coli* networks from DREAM3. Each boxplot represents 40 networks (4 different networks X 10 replicates). **B** same as (**A**) but for the six Yeast networks. **C**–**F** Sorted boxplots of the 4 different *E. coli* networks from DREAM3. Each boxplot represents 10 replicates. Green bar inside each boxplot represents median AUPR value. A complete list of tested MI estimators & GRN inference algo can be found in Additional file 2: Table S2

Next, we examined the performance of these different algorithms with regards to different biological organisms, as *E. coli* and *S. cerevisiae* have distinct distributions of different network motifs (Additional file 2: Figure S11), which may lead to different performance in network inference. For example, the fan-out motif, where one gene regulates two (or more) target genes, is more abundant in *E. coli*, while the cascade motif, where a gene regulates a second gene that in turn regulates a third gene, is more abundant in *S. cerevisiae* [7, 31]. In both cases, the three participating genes exhibit some degree of correlation, yet not all are directly connected. The 10 networks from DREAM3 were divided into four *E. coli* networks (Fig. 5A, C–F, Additional file 2: Fig. S12) and six *S. cerevisiae* networks (Fig. 5B, Additional file 2: Fig. S12, S13). For the combined *E. coli* networks (Fig. 5A), KSG greatly improved the performance of both RL and CMIA algorithms but showed only a modest 6% improvement in performance for CLR. For the combined *E. coli* networks, {KSG,CMIA} achieved a median improvement of 20%, but was second best to {MM,CLR}. The performance comparison of the individual *E. coli* networks (Fig. 5C–F) showed that {KSG,CMIA} was the best performer on three out of four networks. Furthermore, replacing ML with KSG when combined with CLR improved the performance by 10–15% except in the case of DREAM3 Ecoli2-Size100 (Fig. 5F). In the *S. cerevisiae* networks, again KSG improved all algorithms, and most significantly CMIA, and showed a median improvement of 18%. Several replicates did not show any performance improvement, indicating the significance of stochasticity even though all kinetic parameters for each network were identical.

In summary, out of 24 combinations of MI estimators and inference algorithms, the combination {KSG,CMIA} yielded the best median score in 13 out of the 15 networks inspected (except networks DREAM3 Yeast1-Size50 & Ecoli2-Size100, Fig. 5C–F**,** Additional file 2: Figures S13 and S14). Therefore, we conclude that using kNN-based KSG to calculate MI improved the performances of the inference algorithms evaluated in most cases.

### Testing with real expression data from *E. coli*

**Fig. 6:**
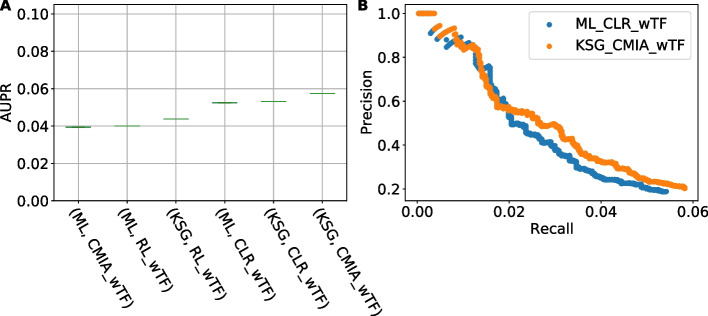
GRN reconstruction performance evaluation for *E*. *coli*. **A** Area Under Precision–Recall curve (AUPR) for six pairs of MI-estimators & Inference algorithms. **B** Two representative precision–recall curves for the top 1000 interactions, where blue dots represents the gold standard in the field {ML,CLR} and orange dots represents our method of choice {KSG,CMIA}

To examine the generality of our method and benchmark on large real networks, we used real *E. coli* expression data (907 different conditions or perturbations) from M3D [32] and documented regulatory interactions from RegulonDB [33]. This new data set added a large real network with 3470 interactions among 1615 genes and 191 transcription factors (TF). AUPR was calculated (Fig. 6A) for six pairs of two MI estimators (ML, KSG) and three inference algorithms (RL, CLR, CMIA), when using prior knowledge about TF, i.e., any gene pair not containing a TF was removed from the final ranked list. Similar to results for synthetic networks, this comparison revealed that using KSG instead of ML as the MI estimator improves the inference algorithm performance. Although the AUPR difference in absolute values is small, when comparing two precision–recall curves for the top 1000 interactions (Fig. 6B) we observe that {KSG,CMIA} added an additional 14 correct interactions (true positives) over {ML,CLR}. Without using prior knowledge about TF all methods gave AUPR close to zero (0.006–0.008). The overall low value of AUPR for large biological networks is a known issue [34] and could be partly explained by the sparseness of the real network relative to the synthetic networks used. The total connectivity of the real symmetric *E. coli* network is {interactions}/({genes}{genes-1}/2) ~ 0.003, while for DREAM3 & 4 in-silico networks it is in the range 0.024–0.141. Additionally, our current incomplete picture of the real network contributes to a higher false positive count which decreases precision and recall.

For the case with known TFs, we also added a kNN scan {1,10} and number-of-bins scan {2,32} for CLR and CMIA (Additional file 2: Figure S15). For kNN scan, this comparison shows a similar trend to Fig. S8, where we observed low variations in AUPR for 1 < k < 16 for the synthetic networks in DREAM3. For number-of-bins scan we also observe small variations in AUPR over a large range of bins. In both cases the small variation observed could be related to the overall low absolute value of AUPR.

### Computational cost

**Fig. 7:**
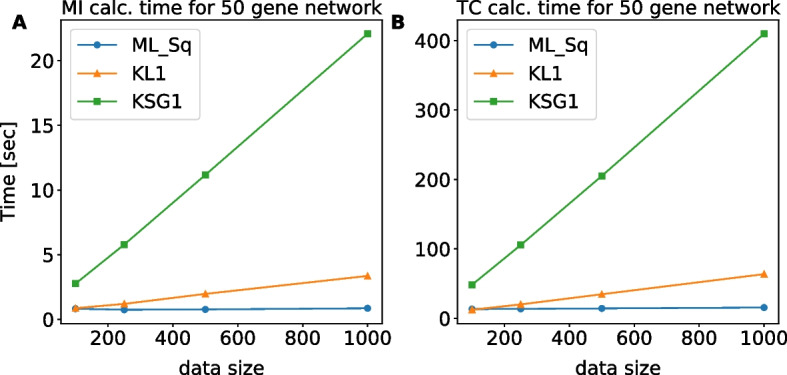
Computation time versus different data sizes for a network of 50 genes. **A** The calculation is performed over 1125 pairs for data sizes of [100, 250, 500, 1000]. **B** The calculation is performed over 19,600 triplets for data sizes as in the left panel

Computational cost is a major concern when applying kNN-based methods. We measured the time required to calculate all the two- and three-way interactions in a 50 gene network (1125 pairs and 19,600 triplets, respectively, after taking symmetry into account) with different data size [100, 250, 500, 1000] for three MI estimation methods: FB-ML, kNN-KL and kNN-KSG. The code for the three estimators was written in Python 3.8, used built-in functions from Numpy v1.19 and Scipy v1.5, and was run on a single core of a desktop [Intel Xeon E5-1620 @ 3.6 GHz]. As seen in Fig. 7 FB-ML was the fastest, as histogram-type calculations have been optimized in Python over the years. FB-ML was also insensitive to data size (in the tested range). While the python-based KSG implementation was most computationally heavy, the time was tractable (under 400 s even for the largest data size (1000) and 3d calculation). The speed could be further boosted by rewriting the code in C/C+ + , similar to what was done by Meyer et al. [35] and Sales et al. [24]. Furthermore, the KD-Tree class of algorithms [36], which was in the main core of this work’s implementation, could greatly benefit from multiple cores or parallel processing. After building the initial tree, distance calculation between neighbors can proceed in parallel, offering 4–16 fold improvement in speed on a current personal computer, depending on the number of available cores.

## Discussion

To date, a plethora of discretization methods, MI estimators, and inference algorithms exist in the literature to reconstruct GRNs. Some common methods are available in the R/Bioconductor package *minet* [35] and in Julia language [37]. In fact, as different methods have certain advantages depending on the investigated scenario and constraints, it is advantageous to consider and compare the performance of different combinations of multiple methods [38].

### kNN-based MI estimator for date discretization/density estimation outperforms fixed-bin-based estimations

Here, we demonstrate that the MI estimator KSG based on kNN yields smaller errors compared to other MI estimation methods using discretized fixed bins in the case of a bi- and tri-variate Gaussian distribution. KSG proves to be robust against different data sizes and correlations as well as the *k* parameter used, unlike FB methods where the parameter used (number of bins) has a large effect on accuracy of the MI estimator. In principle, one can achieve smaller errors using MI based on discretized bins by choosing a different bin number other than the rule of thumb $\sqrt N$, for correlations smaller than 0.9. However, a priori one does not know the correlation strength. In fact, estimating the correlation strength is what one tries to achieve when using MI. We also note that the gene expression profiles of different synthetic networks and real experimental systems could be better described by distributions other than Gaussian. Fortunately, the analytical solution to the mutual information of a few of these distributions can be calculated [39] and will be explored in future work.

Note that in this work we did not compare the performance of another frequently used binning method, adaptive partitioning, which is computationally faster than kNN for large data sets. In brief, adaptive partitioning is a general term referring to three methods that divide the data uniformly between the bins. The first method is equal frequency in which the bin size varies to allow for equal number of data points in each bin. The second method is equiprobable partitioning [21], in which data is ranked and partitioned in the middle, and Pearson chi-square test is used to determine the number of sub-partitions, where the significance level of the chi-square test can be tuned (1–5%) according to the size of the data. This method works well for 1d data, but it has some ambiguity when implemented in higher dimensions in that data points must be ranked according to one of the axes (or more in > 2d), and there are no appropriate rules to rank multidimensional data points. The third method is Bayesian blocks [40], which uses a Bayesian statistics approach to attempt to find the optimal number of bins and their sizes by maximizing a fitness function that depends on those two parameters. While this is a seemingly promising approach, it is unclear how to implement such a method beyond 1d. Because of these reasons, we did not include this binning method in the comparison.

It is important to note that this is not an exhaustive list of available MI estimators. Most rely on more parameters and/or assumptions about the expression data underlying distribution, making them more sensitive and difficult to generalize. Noteworthy are Shrinkage [41] and Dirichlet-based estimators [42] that are designed for small data size, and KDE [14] which requires large data size. In the case of KDE, it is the most computationally costly (relative to estimators used in our benchmark), as it approximates the data distribution using a predefined known distribution (i.e., a Gaussian) with additional user-defined smoothing parameters. This practice can be problematic because in most cases the underlying data distribution is unknown, and experimental data is much sparser than required to achieve results similar to other, simpler methods, such as FB.

### kNN-based MI estimator KSG in combination with CMIA achieves the highest accuracy but may subject to data stochasticity

It is clear from Figs. 3 and 4 that the combination of kSG-based MI estimation and inference algorithm CMIA achieved the highest precision and recall when reconstructing an unknown network. Yet, this combination also showed a large variation in the performance enhancement. As shown in Figs. 4 and 5A–B, we observed that when KSG was combined with CLR or CMIA, a few replicates did not show any performance improvement, or even had a decreased performance indicated by the negative %ΔAUPR value, as indicated by the bottom whisker of the boxplot.

To investigate the source of this variation in the ensemble network plots we inspected different combinations of MI estimators, inference algorithms, data size used, and individual networks (Fig. 5C–F**,** Additional file 2: Fig S13). We found that higher k values (up to k − 15) did not affect the variability in the AUPR results (Additional file 2: Figure S8). However, MI calculation done by KSG exhibited large variations in performance when smaller data size was used as that in the case of 50 gene networks. For example, in Fig. 5C, E, KSG showed a performance enhancement in the range of ~ 25–35% for the three different inference algorithms, but the variability was reduced by half when ML instead of KSG was used. This was also shown in the large variance calculated for KSG for a Gaussian distribution (Additional file 2: Figure S1D, left column). This observation indicates that KSG is more sensitive to stochasticity (intrinsic noise) when data size is smaller than a few hundred points. Our choice of algorithm KSG-1 over KSG-2 (see methods) was intended to keep a low statistical error and thus, low variability. However, using total correlation and two-way mutual information to calculate other measures, such as interaction information (Table 1), can lead to higher errors as the systematic errors might not cancel out as we have demonstrated in this work. Additionally, when using KSG, we set negative values of total correlation and two-way mutual information to zero (due to statistical fluctuations at low correlation values) prior to calculating the other 3d MI quantities. This practice does not change the results for pairs or triplets with highly positive MI values, but in some cases could lead to increased errors as gene pairs with low MI would be ranked differently.

Inference methods that are not purely based on mutual information (thus not included in our comparison), such as KFLR and KFGRNI [43, 44] have an advantage in robustness when using data of small sizes and noisy time-courses, as they can use the temporal data recursively and address the noise explicitly to build a smoother model for the source regulators of each target gene. However, this performance enhancement in robustness comes at the cost of additional model complexity and computation time.

We note that two networks (DREAM3 Yeast1-Size50 & *E.* coli2-Size100) out of the 15 networks investigated showed no performance enhancement when using {KSG, CMIA} compared to the gold standard {ML, CLR} (Fig. 5F, Additional file 2: Fig S13). It is unclear why the performance did not improve in these two cases based on the largely similar statistics of different motifs of the ten networks from DREAM3 (Additional file 3: Table S3). It could be due to a specific sub-structure of this network, but further analysis is needed.

Another important result we observed (Figs. 4 and 5) is that the combination {MM,CLR} achieved higher AUPR for all replicates over {ML,CLR}. This is probably due to the size of the data used, as MM was developed to correct the bias in MI estimation for small data sets. We thus suggest using this combination as the new gold standard of the field when working with similar data sizes and when fixed-binning for data discretization is preferred.

### Using MI based inference algorithms when the true network is unknown

When a true network is not available, it is not possible to use AUPR to select which method to use. As such, in this study we only used AUPR to compare the performance of different methods but did not set an explicit mechanism to threshold between significant and non-significant interactions. Instead, we used the same Z-score threshold concept as that in CLR. In CMIA, as all gene pairs are ranked, one is free to choose an arbitrary Z-score threshold, knowing that there is a tradeoff between setting a high Z-score for high precision (low number of false positive predictions, but also low network discovery) and setting a low Z-score for high recall (higher network discovery, but also high number of false positives). To illustrate this on our method of choice {KSG,CMIA}, we have added a table summarizing the tradeoff between precision and recall for various Z-score values for the real *E. coli* network (see Additional file 3: Table S4). Graphically, the Precision–Recall curves shown in Fig. 6B for the first 1000 gene pairs, show how the precision decreases and the network recall is increased when considering more gene pairs (lower Z-score threshold).

On the other hand, if we have prior knowledge about the underlying network, such as, a list of transcription factors, we can remove interactions that do not include a TF after ranking the interactions by significance. As the network now only contains TF-related interactions, all methods achieved around 50% better AUPR results (Additional file 2: Figure S16) due to the reduction in co-regulated gene pairs (false positives) when non-TF interactions are removed.

### Caveat on time series expression data

Our manuscript focuses on steady-state expression data as it is currently more abundant. Naively, one can use time series data and treat each data point along the time series as though it is a steady-state expression data point for a different perturbation (or experimental condition). To truly benefit from the temporal information, inference algorithms need to be modified as it is not a trivial process [45]. Or new inference algorithms need to be developed [43, 44].

## Conclusions

In summary, we have shown that the kNN-based KSG MI estimator improves the performance of inference algorithms, especially ones that use three-way MI calculations. This result corroborates our observations in comparing MI calculations against the analytical solution of two-way MI of a bi-variate Gaussian distribution and the total correlation of a tri-variate Gaussian distribution. Furthermore, the combination of CMIA and KSG give the overall best performance, and hence should be preferred when precision and recall are more important than speed when reconstructing a GRN. Looking forward, the goal of complete reconstruction of GRNs may require new inference algorithms and probably MI in more than three dimensions.

## Methods

### Calculate mutual information of multiple variables

In Table 1 and Additional file 1: Appendix S1, we summarize the formalism for calculating MI. Shannon’s entropy is the basic building block of MI and represents the randomness of a variable: the more random it is, the more uniformly it is distributed, which gives a higher entropy. For our purposes, X, Y, or Z is a vector (*x*_*1*_*, x*_*2*_*, …, x*_*n*_), (*y*_*1*_*, y*_*2*_*, …, y*_*n*_) or (*z*_*1*_*, z*_*2*_*, …, z*_*n*_) representing a specific gene’s expression profile (data *x, y or z*) under different conditions/perturbations (*n* steady-states) or as a function of time (*n* time points). Two-way MI is defined as the shared (or redundant) information between the two variables X and Y (Table 1) and can be visualized by a Venn diagram (Table 1 right column).

While MI for two variables (genes or dimensions) is readily understood, for three variables or more, new measures arise including Total correlation (TC), Three-way MI (MI3), Interaction Information (II) and Conditional MI (CMI) (Table 1). Unfortunately, the term “three-way MI” has been used loosely in the literature to refer to all four of these measures, and because they represent distinct aspects of statistical dependence, in the context of GRN reconstruction, this can lead to different realizations. Unlike other MI quantities, Interaction-Information is hard to visualize using a Venn diagram, as it can have both positive and negative values. It is common to regard negative II as “Redundancy”, the shared information between all variables, and positive II as “Synergy”. Synergy can be interpreted as new information gained on the dependence between two variables {X,Y} when considering the contribution of a third variable {Z} on either {X} or {Y} versus without considering it, or mathematically: II = CMI(X;Y|Z)-MI(X;Y).

To calculate the marginal and joint entropies of two variables (X and Y), we first need to know the probability of each data point. For discrete data, we can approach the underlying probability *p(x)* by calculating the frequency $\left( {f_{x} = \frac{{N_{x} }}{{N_{Tot} }}} \right)$ where N_x_ is the number of data points with value *x*, and N_Tot_ is the total sample size. For the continuous data case, the calculation is more complex. Although Shannon extended his theory for continuous data by replacing the summation with integrals, it is common practice in the field to discretize the data first so one can work with the discrete formalism (Table 1). The simplest discretization method is to use fixed (width) binning (FB) (Fig. 1A), but the optimal binning choice depends on the shape of the distribution and data size. For normally distributed data, the rule of thumb is to use the square-root of the data size as the number of bins.

#### k-nearest-neighbor (kNN)

Other than evaluating the probability densities to calculate mutual information, Kozachenko and Leonenko (KL) calculated the marginal and joint entropies (and the MI by summation) from the mean distance to the kth-nearest neighbor [25]. To minimize errors when combining entropies of different dimensions, Kraskov et al. calculate the MI directly [22]. KSG developed two algorithms, I^(1)^ and I^(2)^ (hereafter, KSG-1 and KSG-2), to minimize errors when estimating MI compared to previous methods. We chose KSG-1 (defined below as MI_KSG_) as it gives slightly smaller statistical error (dispersion). Note that although KSG-1 gives relatively larger systematic errors than KSG-2, these systematic errors do not change the ranking of the output values (from high to low), which is what we use in downstream analysis. An additional note is that using kNN can lead to negative values for mutual information, which contradicts Shannon’s theorem. Negative values are caused by statistic fluctuations when there is no correlation between variables. Therefore, in such a situation, we set negative values to zero (except for Interaction Information, where it is meaningful). To calculate MI using the KSG method, we use the following formulas:

$$MI_{KSG} \left( {X;Y} \right) = \psi \left( k \right) - \langle \psi \left( {n_{x} + 1} \right) + \psi \left( {n_{y} + 1} \right) \rangle + \psi \left( N \right)$$

$$TC_{KSG} \left( {X;Y;Z} \right) = \psi \left( k \right) - \langle \psi \left( {n_{x} } \right) + \psi \left( {n_{y} } \right) + \psi \left( {n_{z} } \right) \rangle + 2 \cdot \psi \left( N \right)$$

where ψ(x) is the digamma function, N is the number of data points, n_i_ is the number of points x_j_ whose distance from x_i_ is less than ε(i)/2, and ε(i)/2 is the distance from u_i_ = (x_i_,y_i_,z_i_) to its kth neighbor, as illustrated in Fig. 1a of [22]

### In-silico GRN Inference comparison

**Fig. 8:**
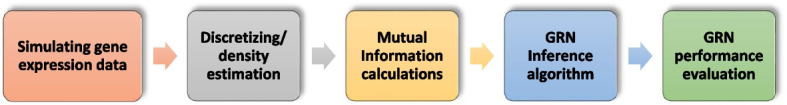
The different steps for evaluating GRN inference performance

MI calculations are used to infer interactions between genes to reconstruct the underlying GRN structure. To test the performance of different methods, we followed the methodology of the in-silico network inference challenges of the Dialogue for Reverse Engineering Assessments and Methods (DREAM) competitions DREAM3/4 [27] as depicted in Fig. 8.

**Fig. 9:**
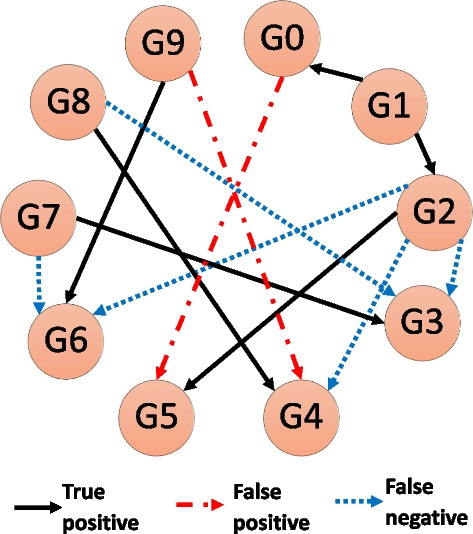
A schematic GRN inference example. The true network contains 10 genes (a.k.a. nodes), and 11 interactions (or edges). The prediction algorithm correctly predicted 6 times (True positive), missed 5 interactions (False negative), and predicted 2 interactions that did not exist (False positive)

1. *Simulating gene expression data*—we used GeneNetWeaver [29] to generate steady-state and time-series gene expression datasets for realistic in-silico networks of sizes of 50, and 100 genes containing various experimental conditions (knockouts, knockdowns, multifactorial perturbation, etc.). GeneNetWeaver uses a thermodynamic model to quantify mRNA transcription and regulation with added molecular and experimental noise.
2. *Discretizing/density estimation*—To handle the continuous expression data, we chose either:
  - Density estimation by fixed binning. We used the common practice sqrt(n), where n = number of data points (in our case, different experimental conditions), as the number of bins.
  - Density estimation by k-Nearest Neighbor (kNN). We chose k = 3 as a good compromise between precision and computation cost as discussed in the previous section.
3. *Mutual Information estimation*—Depending on our previous selection, we chose between several MI estimators:
  - For the fixed-bin discretizing method, we used either Shannon’s formula (also referred to as *Maximum Likelihood,* ML) or *Miller–Madow* (MM) estimator.
  - For kNN we used either KL or KSG formulas for MI.
4. *GRN inference algorithms*—We used popular algorithms in the field that use either only two-way MI or both two- and three-way MI to infer undirected network structure by sorting predicted interacting gene pairs from most probable to least probable. Each algorithm starts with a MI matrix containing calculation for all possible pairs (some use all possible triplets) and applies different rules to filter results and sort the gene pairs (see summary below). We used the same MI matrices for a fair comparison between the inference algorithms. The following algorithms were used in our comparison:
  - Relevance Network (RL)—Gene pairs are sorted according to their MI(X;Y) value from highest to lowest, and a threshold applied to truncate non-significant results [10]. By not setting a threshold we maximize AUPR (see below) and remove a free parameter from the performance evaluation.
  - Algorithm for the Reconstruction of Accurate Cellular Networks (ARACNE)—Same as RL with the addition of Data Processing Inequality (DPI), which means for every three genes MI is calculated for each pair and the pair with the lowest MI is removed if the difference is larger than some threshold [11]. In our implementation, we set the threshold to zero, so we always removed the lowest interacting pair, similar to the implementation in *Minet* [35]. On the other extreme, when the threshold is high, all pairs are kept and ARACNE is identical to RL.
  - Context Likelihood of Relatedness (CLR)—Background correction is performed by calculating Z-score for the MI of each gene interacting with all other genes, and then gene pairs are sorted by their mutual Z-score [12]. A threshold based on Z-score value is than used to remove non-significant interactions. Similar to RL we did not set a significance threshold. We also did not use B-spline smoothing in the density estimation step in accordance with the implementation in the R-package *Minet* [35].
  - Synergy Augmented CLR (SA-CLR)—Same as CLR, with the difference that now the highest Interaction-Information term is added to MI prior to performing the background correction [17]
  - Conditional Mutual Information Augmentation (CMIA)—Similar to SA-CLR but we used conditional mutual information instead of interaction-information.
  - Luo et al. MI3 (hereafter CMI2rt)—We assumed two regulators for each target gene, and for each target gene we searched for the best {R1,R2} pair that maximizes: CMI(T;R1|R2) + CMI(T;R2|R1) [14]
5. *GRN performance evaluation*—To evaluate the performance of common algorithms in the field, we used known (true) synthetic networks and counted the number of true and false positives (TP and FP respectively) predictions as well as true and false negative (TN and FN respectively) (Fig. 9). This allowed us to plot precision (Precision = TP/(TP + FP)) versus recall (Recall = TP/(TP + FN)) and calculate the area under precision–recall curve (AUPR). As biological networks are sparse on edges, AUPR is considered a better metric than AUROC (area under the receiver operating characteristic curve, which is the false positive rate FPR = FP/(FP + TN vs. recall) as mentioned elsewhere [46].

## Supplementary Information

**Additional file 1**. **Appendix S1**: Mutual Information overview. **Appendix S2**: Analytical solution for a multivariate Gaussian distribution. **Appendix S3**: Miller–Madow correction to Shannon’s entropy.

**Additional file 2**. **Figure S1**: 100 replicates of two-way mutual information (MI2) & total correlation (TC) for multivariate gaussian dist. With sample size = {100,1K,10K}, correlation = {0.3,0.6,0.9}. **Figure S2–S4**: boxplots of percent error of three different mutual information estimators for 100 replicates of tri-variate gaussian dist. **Figure S5**: boxplots of percent error of two-way mutual information calculated based on kNN methods for 100 replicates of bi-variate gaussian dist. With sample size = {100,1K,10K}, correlation = {0.3,0.6,0.9}. **Figure S6**: boxplots of percent error of Total Correlation calculated based on kNN methods for 100 replicates of tri-variate gaussian dist. **Figure S7**: Precision–recall curves of six MI-Inference algorithms of five different synthetic networks from DREAM4. **Figure S8**: Area Under Precision–Recall curve (AUPR) versus different number of bins or k-neighbors for networks from DREAM3. **Figure S9**: AUPR for randomized data versus true data. **Figure S10**: Comparison of different combinations of MI estimators and inference algorithms used in this work with PIDC and Grnboost2 for networks of different sizes and types. **Figure S11**: Common 3-node network motifs. **Figure S12**: AUPR Performance comparison of GRN reconstruction for different in silico networks modeled from E. coli & Yeast. **Figure S13**: Sorted boxplots of percentage AUPR difference (increase or decrease) relative to the gold standard combination [ML,CLR] for different combinations of MI estimator and GRN inference algorithm for the 6 different Yeast networks from DREAM3. **Figure S14**: Sorted boxplots of percentage AUPR difference (increase or decrease) relative to the gold standard combination [ML,CLR] for different combinations of MI estimator and GRN inference algorithm for the 5 different networks of 100 genes from DREAM4. **Figure S15**: Area Under Precision–Recall curve (AUPR) versus different number of bins or k-neighbors for real E. coli data. **Figure S16**: AUPR comparison of different combinations of MI estimators and inference algorithms when non-TF-containing interactions are removed from the networks.

**Additional file 3.** **Table S1**: Median AUPR and %ΔAUPR (AUPR_relative) values for different combinations of MI estimator and GRN inference algorithm for different network sizes. **Table S2**: Median AUPR and %ΔAUPR (AUPR_relative) values for different combinations of MI estimator and GRN inference algorithm for different organisms. **Table S3**: Characteristics of the 10 synthetic networks from DREAM3 and statistics of the different 3-node network motifs extracted. **Table S4**: precision and recall for various Z-score values for the real *E*. *coli* network.

## Data Availability

The software GeneNetWeaver used to generate the datasets in the current study is available in the GitHub repository, https://github.com/tschaffter/genenetweaver. The code and scripts used for analysis and to generate the plots in the current study are available in the GitHub repository, https://github.com/XiaoLabJHU/GRN_Inference. The GRN inference pipeline implemented here is modular, one can use specific functions to calculate MI quantities based on kNN and integrate the output matrix into a different inference algorithm than the ones implemented in this work.

## Abbreviations

GRN: Gene regulatory network
ODE: Ordinary differential equation
MI: Mutual information
PDF: Probability density function
FB: Fixed (width) binning
AP: Adaptive partitioning
kNN: k-nearest neighbor
KDE: Kernel density estimator
CLR: Context likelihood of relatedness
CMIA: Conditional mutual information augmentation
KSG: Kraskov–Stoögbauer–Grassberger
RL: Relevance networks
ARACNE: Algorithm for the Reconstruction of Accurate Cellular Networks
SA-CLR: Synergy-augmented CLR
ML: Maximum likelihood
MM: Miller–Madow
KL: Kozachenko–Leonenko
TC: Total correlation
MI3: Three-way MI
II: Interaction information
CMI: Conditional mutual information
DREAM: Dialogue for reverse engineering assessments and methods
AUPR: Area under precision–recall curve
CMI2rt: Luo et al. inference algorithm named MI3
DPI: Data processing inequality
TF: Transcription Factor
