# Supplementary material for: Gene regulation network inference using k-nearest neighbor-based mutual information estimation: revisiting an old DREAM
Source: BMC Bioinformatics. 2023 Mar 6;24:84. doi: 10.1186/s12859-022-05047-5 (PMC9990267; doi:10.1186/s12859-022-05047-5)
Supplement: Supplementary file 1 — Additional file 1. Appendix S1: Mutual Information overview. Appendix S2: Analytical solution for a multivariate Gaussian distribution. Appendix S3: Miller–Madow correction to Shannon’s entropy. [file 12859_2022_5047_MOESM1_ESM.docx]

**Additional file 1: Supplementary information Appendix 1-3**

**Appendix S1**: Mutual Information overview

Introduction to Information Theory

Information Theory [1] main use outside the world of communication is to determine the "similarity" between different sets of variables and help determine if there is any correlation between them whether it is linear or non-linear.

Shannon’s Entropy

X is a discrete random variable (i.e. X ≡ {x_1_, x_2_, ..., x_n_}), this could be the outcomes of flipping a coin multiple times or rolling a dice. Shannon [1] defined the "uncertainty" of X as the Entropy of X, H(X). Entropy is a non-negative quantity (H(X) ≥ 0), it is maximal where all possible outcomes (more than one) have the same probability (in other words, a uniform probability distribution function). For example, flipping a fair coin or rolling a dice (the entropy is maximal but not equal for those two cases). On the other hand, if our variable X has only one possible value than there is no uncertainty, and the entropy equals zero. In some places, entropy is also referred to as "self-information" (see below section on Mutual Information).

Conditional and Joint Entropy

Following the same logic, the joint entropy H(X,Y), is defined as the uncertainty of the pair X, Y. And the conditional entropy H(X|Y), is the uncertainty of X given Y. We can represent their relationship in the following formula (mathematical proofs can be found in [2]):

H(X,Y) = H(X) + H(Y|X) = H(Y) + H(X|Y)

We can extend the above relationship to 3 variables:

H(X,Y,Z) = H(X) + H(Y,Z|X) = H(X) + H(Y|X) + H(Z|Y,X)

For *n* variables (Chain rule for entropy) [2]:

$$H\left( X_{1},X_{2},\ldots,X_{n} \right)=\sum_{i=1}^{n} H\left( X_{i}|X_{i-1},\ldots,X_{1} \right)$$

Mutual Information

We can now define the Mutual Information (MI) shared by X and Y as:

$I\left( X;Y \right)=H\left( X \right)-H\left( X|Y \right)=H\left( Y \right)-H\left( Y|X \right)=H\left( X \right)+H\left( Y \right)-H\left( X,Y \right)$ (1)

This is also referred to as "information redundancy" or "Reduction of uncertainty" of X thanks to Y (or vice versa). MI is a symmetric (I(X;Y) = I(Y;X)) and non-negative (I(X;Y) ≥ 0) quantity. It is zero only if X and Y are independent (H(X,Y) = H(X) + H(Y)). We also get that self-information equals the entropy (I(X;X) = H(X)). Summarizing the above, we get a range for MI: 0 ≤ I(X;Y) ≤ max{H(X),H(Y)}.

Three-Dimensional Mutual Information

In 1954, McGill [3] extended Shannon’s work to the case of two sources {X_1_,X_2_} and one receiver Y (or vice versa), by simply changing X in Eq. (1) with {X_1_,X_2_}:

I(X_1_,X_2_;Y) = H(X_1_,X_2_) − H(X_1_,X_2_|Y) = H(X_1_,X_2_) + H(Y) − H(X_1_,X_2_,Y)

Naturally, this can be extended to n variables, where we can measure the mutual information between a group of n − 1 variables (treated as a single vector) and a target. However, this doesn’t allow us to evaluate the individual gain (or loss) of information by each individual source, but this can be solved by comparing calculations with different number of sources.

Interaction Information

McGill also defined the Interaction-Information (II), which is a symmetric quantity:

II(X_1_· X_2_·Y ) = I(X_1_; Y | X_2_) − I(X_1_; Y ) = I(X_2_; Y | X_1_) − I(X_2_; Y ) = I(X_1_; X_2_|Y ) − I(X_1_; X_2_) (2)

Where the Conditional Mutual Information (CMI) of X_1_ and X_2_ given Y is defined by:

I(X_1_; X_2_|Y ) = H(X_1_|Y ) − H(X_1_| X_2_, Y ) = −H(Y ) + (H(X_1_, Y ) + H(X_2_, Y )) − H(X_1_, X_2_, Y ) (3)

It is important to note, that the RHS of Eq. (3) represent only two out of many possible combinations of entropy terms. We can also write the interaction information as an expansion of entropy terms:

II(X_1_ · X_2_· Y ) = − H(X_1_) − H(X_2_) − H(Y ) + H(X_1_, X_2_) + H(X_1_, Y ) + H(X_2_, Y ) − H(X_1_, X_2_, Y )

We can now write the three-dimensional mutual information using the two-dimensional MI and the interaction-information:

I(X_1_, X_2_; Y ) = I(X_1_; Y ) + I(X_2_; Y ) + II(X_1_ · X_2_· Y ) (4)

I(X_1_, X_2_; Y ) = I(X_1_; Y | X_2_) + I(X_2_; Y | X_1_) − II(X_1_ · X_2_· Y )

We can plug Eq. (2) into the three-dimensional MI equation above Eq. (4), to get:

I(X_1_, X_2_; Y ) = I(X_2_; Y ) + I(X_1_; Y | X_2_) = I(X_1_; Y ) + I(X_2_; Y | X_1_) = I(X_1_; Y ) + I(X_2_; Y ) + I(X_1_; X_2_|Y ) − I(X_1_; X_2_)

We can expand the MI to *n* sources (Chain rule for information [2]):

$$I\left( X_{1},X_{2},\ldots,X_{n};Y \right)=H\left( X_{1},X_{2},\ldots,X_{n} \right)-H\left( X_{1},X_{2},\ldots,X_{n}|Y \right)=\sum_{i=1}^{n} I\left( X_{i};Y|X_{i-1},\ldots,X_{1} \right)$$

Total Correlation

Total Correlation (TC) is another frequently used term (also referred sometime as redundancy or MI^[[1]](#footnote-1)^) that was first shown by McGill [3] but was coined and further developed by Watanabe in 1960 [4].

$$TC\left( X_{1},X_{2},\ldots,X_{n} \right)=\sum_{i=1}^{n} H\left( X_{i} \right)-H\left( X_{1},X_{2},\ldots,X_{n} \right)$$

By adding and subtracting the same joint entropy terms, we can rewrite the TC using MI terms (see Appendix A of [5]):

TC(X_1_, X_2_, ..., X_n_) = I(X_1_; X_2_) + I(X_1_, X_2_; X_3_) + ... + I(X_1_, ..., X_n-1_; X_n_)

Uniqueness, Redundancy and Synergy

"Redundancy" and "Synergy" are common terms in the field of information theory, yet they lack common definition, and so create a lot of confusion as different definitions exists. We can start discussing their meaning when looking into the relationship between three variables or more (i.e. two sources X_1_, X_2_ and a target Y). In the most intuitive way, we can define redundancy as the portion of information both X_1_ and X_2_ share in common about Y, and synergy as information we gain (or emerges) about Y from inspecting X_1_ and X_2_ together, rather than separately. Following the same line of thought "Uniqueness" can be viewed as the information only X_1_ brings about Y or only what X_2_ brings about Y. Using the terms we defined in the previous section, we can write: CMI = Uniqueness, II = Redundancy if II < 0, and Synergy if II > 0.

Formalism for Discrete Variables

For variables X and Y, we can construct a space X − Y where each point corresponds to each pair {x, y}. We can generate any ensemble X − Y by assigning a joint probability P(x,y). Where

$$\sum_{X} \sum_{Y} P\left( x,y \right)=1$$

The probability distribution P(x) (also called "marginal") can be defined in terms of P(x,y) by

$$P\left( x \right)≝\sum_{Y} P\left( x,y \right)$$

The conditional probability distribution p(y|x) is defined as

$$P\left( y|x \right)≝\frac{P\left( x,y \right)}{P\left( x \right)}$$

For three variables, we can define the conditional probability distribution P(x|y,z) as

$$P\left( x|y,z \right)≝\frac{P\left( x,y,z \right)}{P\left( y,z \right)}$$

If $P\left( x|y,z \right)$is independent of any pair *y, z* ($P\left( x|y,z \right)=P\left( x \right)$) than X is independent of Y, Z and we can write $P\left( x,y,z \right)=P\left( x \right)P\left( y,z \right)$

Shannon’s Entropy: $H\left( X \right)=-\sum_{x} p\left( x \right)\log p\left( x \right)$

Joint Entropy: $H\left( X,Y \right)=-\sum_{x} \sum_{y} p\left( x,y \right)\log p\left( x,y \right)$

Conditional Entropy: $H\left( X|Y=y \right)=-\sum_{x} p\left( x|y \right)\log p\left( x|y \right)$

$$H\left( X|Y \right)=\sum_{y} p\left( y \right)H\left( X|Y=y \right)=\sum_{y} p\left( y \right)\sum_{x} p\left( x|y \right)\frac{1}{\log p\left( x|y \right)}$$

Information provided by *y_i_* about *x_k_* is defined by:

$$I\left( x_{k};y_{i} \right)≝\log\frac{P\left( x_{k}|y_{i} \right)}{P\left( x_{k} \right)}=\log\frac{P\left( x_{k}|y_{i} \right)P\left( y_{i} \right)}{P\left( x_{k} \right)P\left( y_{i} \right)}=\log\frac{P\left( x_{k},y_{i} \right)}{P\left( x_{k} \right)P\left( y_{i} \right)}$$

This can be positive or negative, depending on the probability of occurring together vs. separately.

Mutual Information (MI): $I\left( X;Y \right)=-\sum_{x} \sum_{y} p\left( x,y \right)\log\frac{p\left( x,y \right)}{p\left( x \right)p\left( y \right)}$

Conditional Mutual Information (CMI): $I\left( X;Y|Z \right)=-\sum_{z} p\left( z \right)\sum_{x} \sum_{y} p\left( x,y|z \right)\log\frac{p\left( x,y|z \right)}{p\left( x|z \right)p\left( y|z \right)}$

**Appendix S2**: Analytical solution for a multivariate Gaussian distribution

Shannon [1] showed that the entropy term of a multivariate Gaussian distribution is given by:

$$H\left( X \right)=\frac{1}{2}\log\left[ \left( 2\pi e \right)^{n}\left| COV \right| \right]$$

Where |COV| represents the covariance matrix. For simplicity we set all the correlations between variables to be equal to ρ.

As all MI quantities can be calculated by their entropy components, we have:

$$MI\left( X;Y \right)=H\left( X \right)+H\left( Y \right)-H\left( X,Y \right)=-\frac{1}{2}\log(1-\rho^{2})$$

$$TC\left( X;Y;Z \right)=H\left( X \right)+H\left( Y \right)+H(Z)-H\left( X,Y,Z \right)=-\frac{1}{2}\log(1-3\cdot\rho^{2}+2\cdot\rho^{3})$$

**Appendix S3**: Miller-Madow correction to Shannon’s entropy

Due to the logarithmic nature of Shannon’s entropy:

$$H^{Shan}\left( X \right)=-\sum_{x} p\left( x \right)\log\left( p\left( x \right) \right)$$

Under or overestimating p(x) by the same value gives different errors on the entropy calculation, leading to bias (downwards). Miller and Madow proposed to correct the bias in Shannon’s entropy by adding the asymptotic bias term [6]:

$$H^{MM}=H^{Shan}+\frac{\left\{ non\_empty\_bins \right\}-1}{2N}$$

Where N is equal to the data size.

Two-way mutual information and higher dimension measures can be calculated by summation of entropies, for example in the case of two-way MI:

$${MI}^{MM}\left( X;Y \right)=H^{MM}\left( X \right)+H^{MM}\left( Y \right)-H^{MM}\left( X,Y \right)$$

**References**

1. Shannon, C. E. (1948). A Mathematical Theory of Communication. *Bell System Technical Journal*, *27*(4), 623–656. <https://doi.org/10.1002/j.1538-7305.1948.tb00917.x>
2. Cover, T. M., & Thomas, J. A. (2005). Elements of Information Theory. In *Elements of Information Theory*. John Wiley and Sons. <https://doi.org/10.1002/047174882X>
3. McGill, W. Multivariate information transmission. Transactions of the IRE Professional Group on Information Theory 4, 93–111 (Sept. 1954).
4. Watanabe, S. Information Theoretical Analysis of Multivariate Correlation. IBM Journal of Research and Development 4, 66–82 (Jan. 1960).
5. Timme, N., Alford, W., Flecker, B. & Beggs, J. M. Synergy, redundancy, and multivariate information measures: An experimentalist’s perspective. Journal of Computational Neuroscience 36, 119–140 (2014).
6. Miller, G.A. (1955). Note on the bias of information estimates. Information Theory in Psychology; Problems and Methods, II-B, 95–100

1. This confusion is mainly due to the fact than in 2d they are all expressed the same but for higher dimensions (n > 2) they are different. [↑](#footnote-ref-1)
