# Supplementary material for: Gene regulation network inference using k-nearest neighbor-based mutual information estimation: revisiting an old DREAM
Source: BMC Bioinformatics. 2023 Mar 6;24:84. doi: 10.1186/s12859-022-05047-5 (PMC9990267; doi:10.1186/s12859-022-05047-5)
Supplement: Supplementary file 2 — Additional file 2. Figure S1: 100 replicates of two-way mutual information (MI2) & total correlation (TC) for multivariate gaussian dist. With sample size = {100,1K,10K}, correlation = {0.3,0.6,0.9}. Figure S2–S4: boxplots of percent error of three different mutual information estimators for 100 replicates of tri-variate gaussian dist. Figure S5: boxplots of percent error of two-way mutual information calculated based on kNN methods for 100 replicates of bi-variate gaussian dist. With sample size = {100,1K,10K}, correlation = {0.3,0.6,0.9}. Figure S6: boxplots of percent error of Total Correlation calculated based on kNN methods for 100 replicates of tri-variate gaussian dist. Figure S7: Precision–recall curves of six MI-Inference algorithms of five different synthetic networks from DREAM4. Figure S8: Area Under Precision–Recall curve (AUPR) versus different number of bins or k-neighbors for networks from DREAM3. Figure S9: AUPR for randomized data versus true data. Figure S10: Comparison of different combinations of MI estimators and inference algorithms used in this work with PIDC and Grnboost2 for networks of different sizes and types. Figure S11: Common 3-node network motifs. Figure S12: AUPR Performance comparison of GRN reconstruction for different in silico networks modeled from E. coli & Yeast. Figure S13: Sorted boxplots of percentage AUPR difference (increase or decrease) relative to the gold standard combination [ML,CLR] for different combinations of MI estimator and GRN inference algorithm for the 6 different Yeast networks from DREAM3. Figure S14: Sorted boxplots of percentage AUPR difference (increase or decrease) relative to the gold standard combination [ML,CLR] for different combinations of MI estimator and GRN inference algorithm for the 5 different networks of 100 genes from DREAM4. Figure S15: Area Under Precision–Recall curve (AUPR) versus different number of bins or k-neighbors for real E. coli data. Figure S16: AUPR comparison of different com [file 12859_2022_5047_MOESM2_ESM.docx]

**Additional file 2: Supplementary figures S1-18**

**Figure S1**: 100 replicates of two-way mutual information (MI2) & total correlation (TC) for multivariate gaussian dist. With sample size = {100,1K,10K}, correlation = {0.3,0.6,0.9}. (A) MI2 with natural log base calculated using Maximum Likelihood with fixed width binning (FB), where the shaded area represents mean +/- 2std. (B) MI2 based on KSG k-nearest-neighbor (KNN). (C) TC based on FB. (D) TC based on kNN

C

A

| 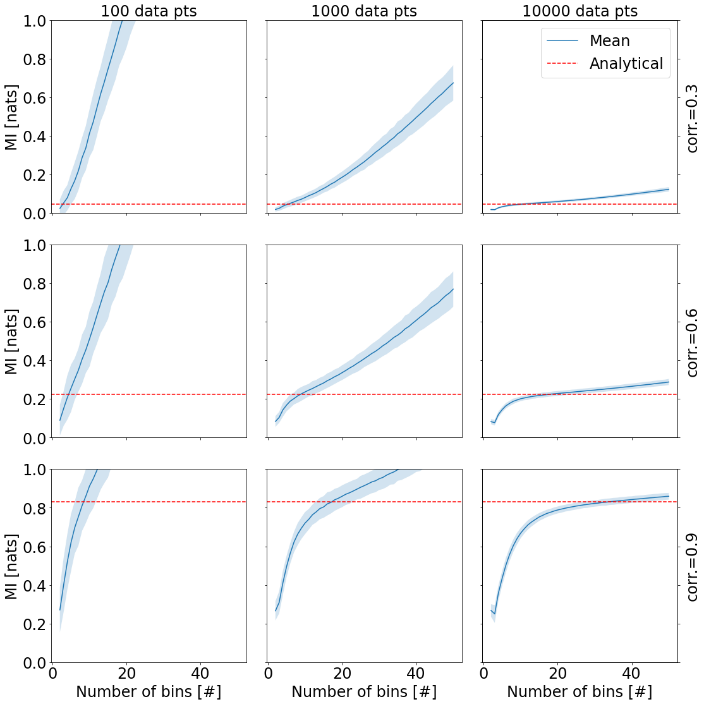  B  D | 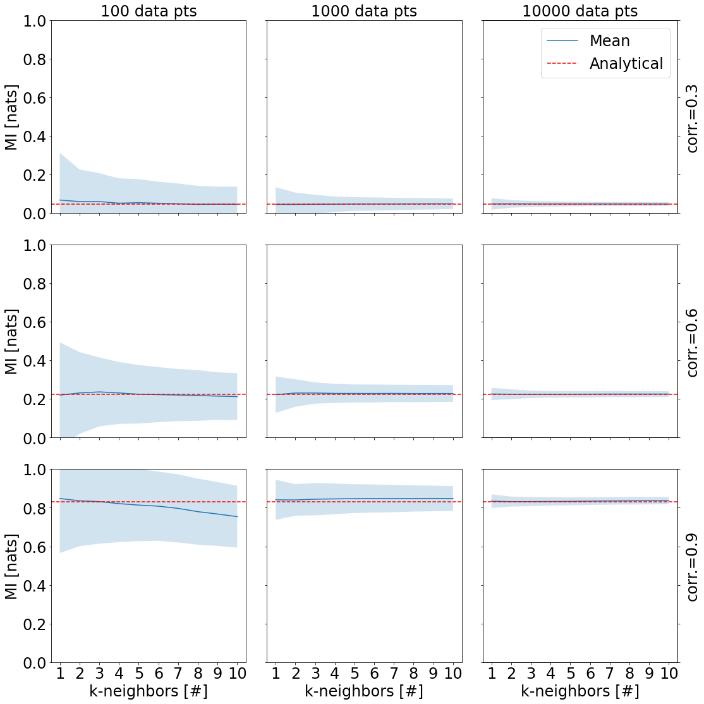 |
| --- | --- |
| 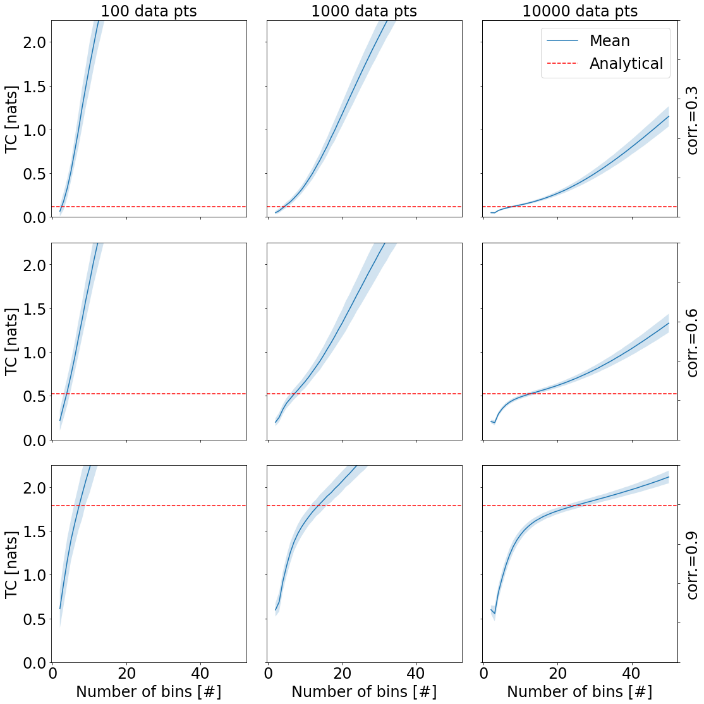 | 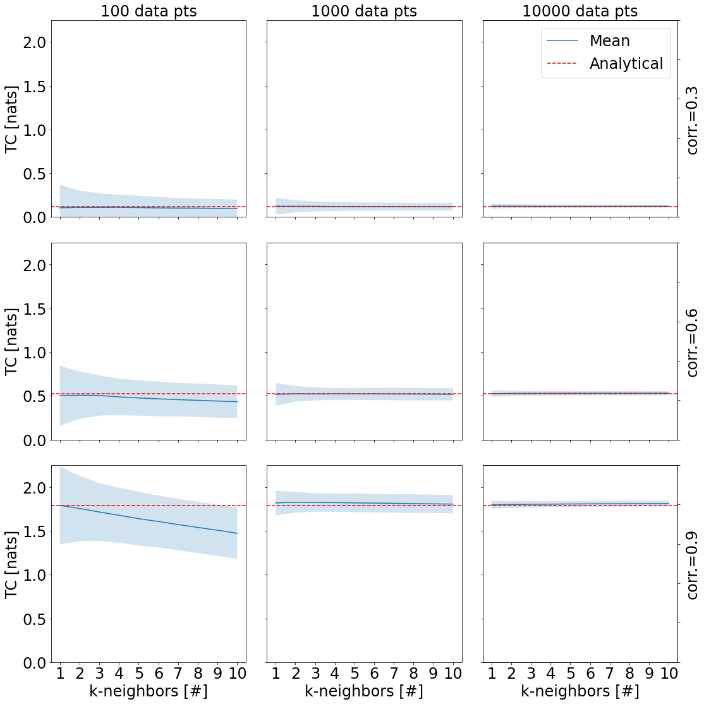 |

**Figure S2**: boxplots of percent error of three different mutual information estimators for 100 replicates of tri-variate gaussian dist. With columns representing sample size = {100,1K,10K}, and rows the correlation = {0.3,0.6,0.9}. 9 subplots showing percent error for Interaction Information (II) for 3 different methods: Sqrt(N)=Shannon’s MI with fixed width binning (number of bins is determined by square-root), MM_Sq=Miller-Madow formula for MI with square-root for the number of bins, kNN3=KSG formula for MI with k=3. **
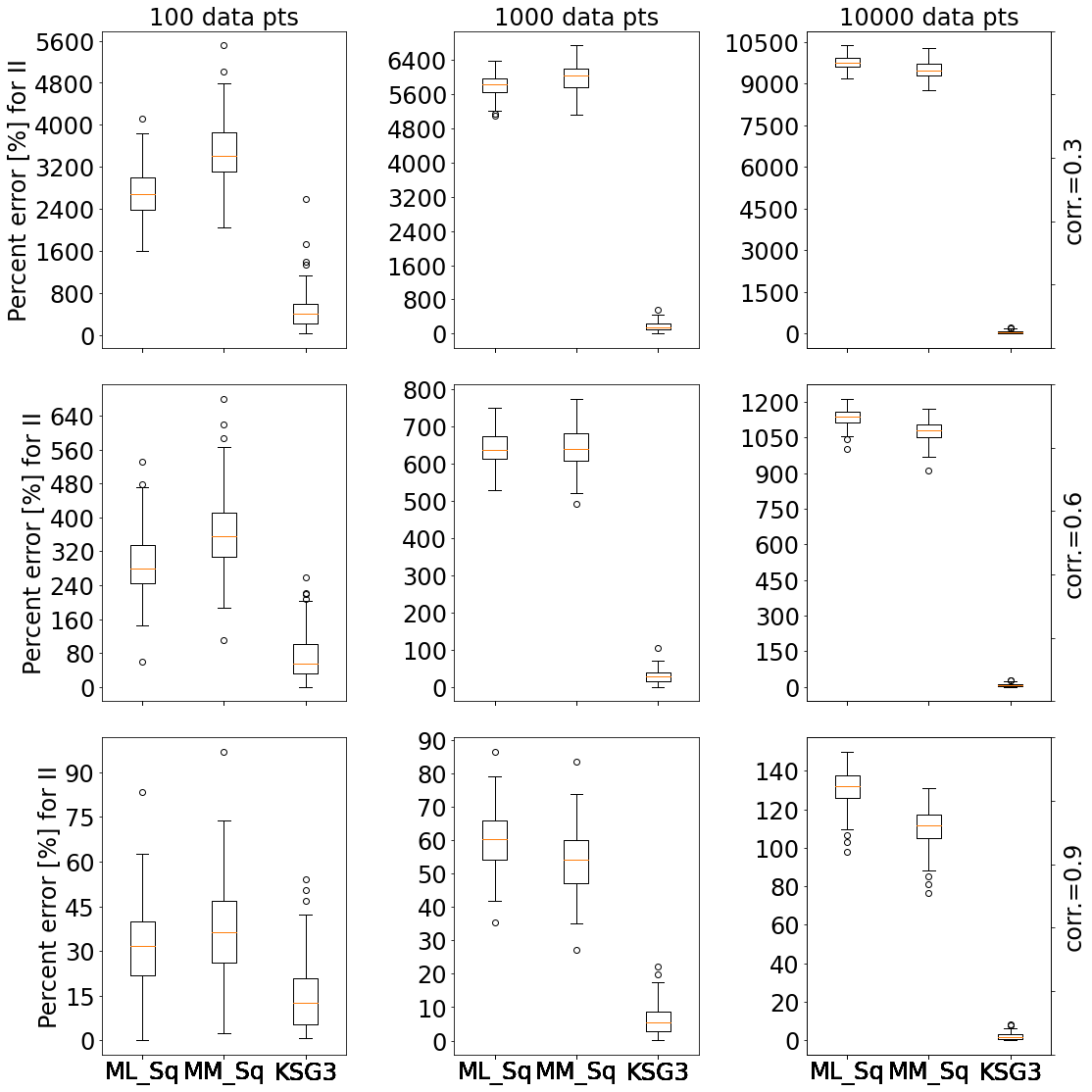
**

**Figure S3**: boxplots of percent error of three different mutual information estimators for 100 replicates of tri-variate gaussian dist. With columns representing sample size = {100,1K,10K}, and rows the correlation = {0.3,0.6,0.9}. 9 subplots showing percent error for Conditional Mutual Information (CMI) for 3 different methods: Sqrt(N)=Shannon’s MI with fixed width binning (number of bins is determined by square-root), MM_Sq=Miller-Madow formula for MI with square-root for the number of bins, kNN3=KSG formula for MI with k=3.

**
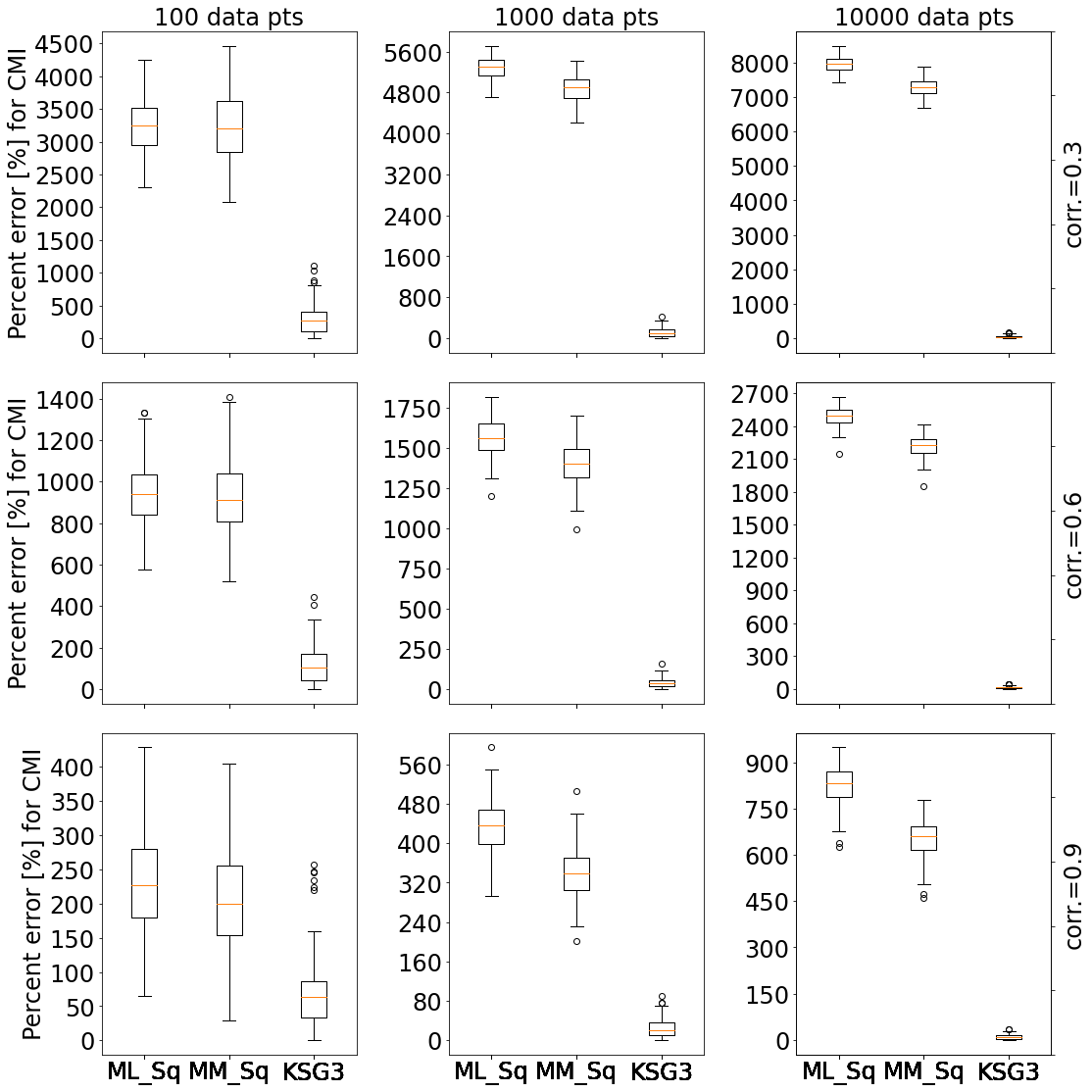
**

**Figure S4**: boxplots of percent error of three different mutual information estimators for 100 replicates of tri-variate gaussian dist. With columns representing sample size = {100,1K,10K}, and rows the correlation = {0.3,0.6,0.9}. 9 subplots showing percent error for Three-way Mutual Information (MI3) for 3 different methods: Sqrt(N)=Shannon’s MI with fixed width binning (number of bins is determined by square-root), MM_Sq=Miller-Madow formula for MI with square-root for the number of bins, kNN3=KSG formula for MI with k=3.

**
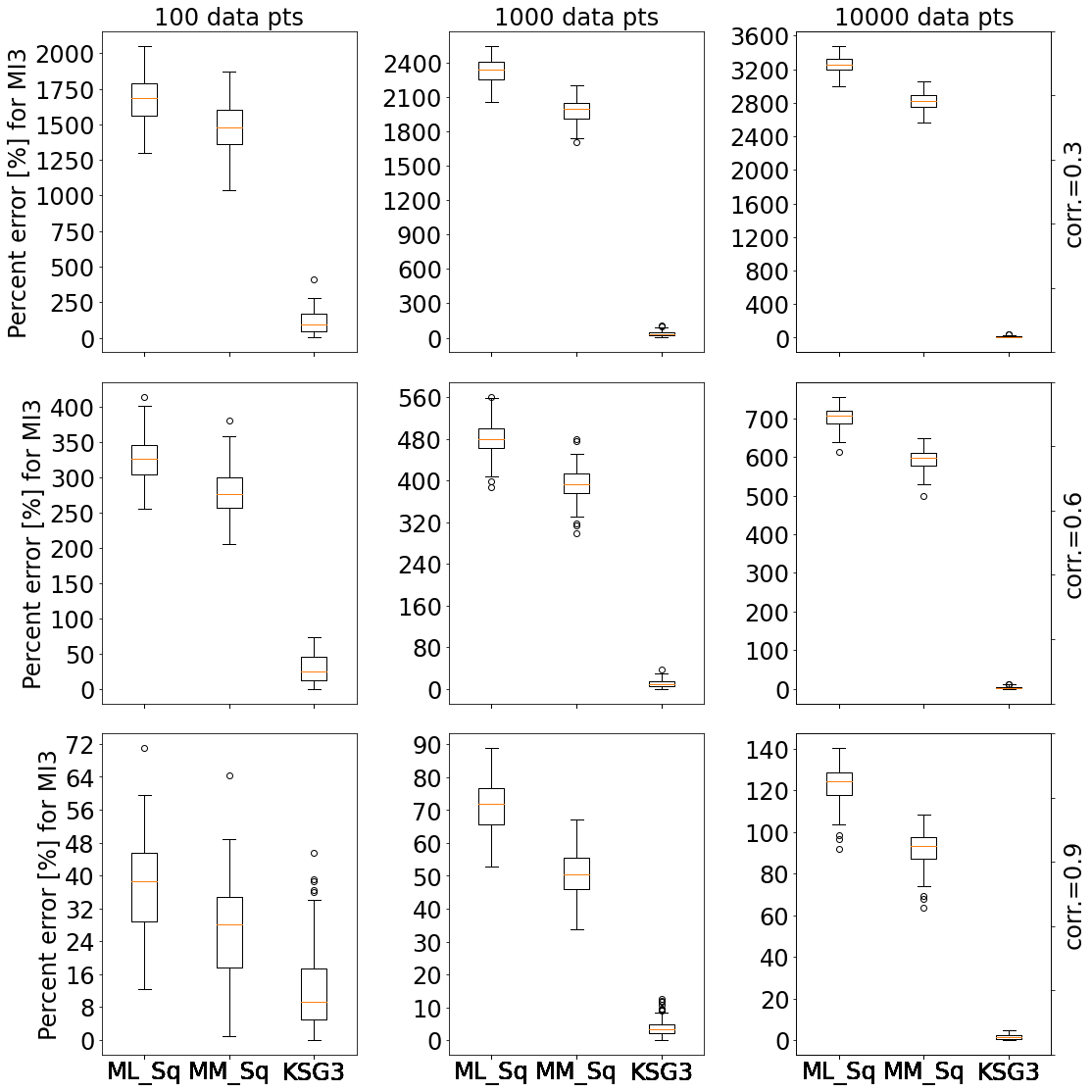
**

**Figure S5**: boxplots of percent error of two-way mutual information calculated based on kNN methods for 100 replicates of bi-variate gaussian dist. With sample size = {100,1K,10K}, correlation = {0.3,0.6,0.9}. We compare KL and KSG methods for k=1,3,10.

**
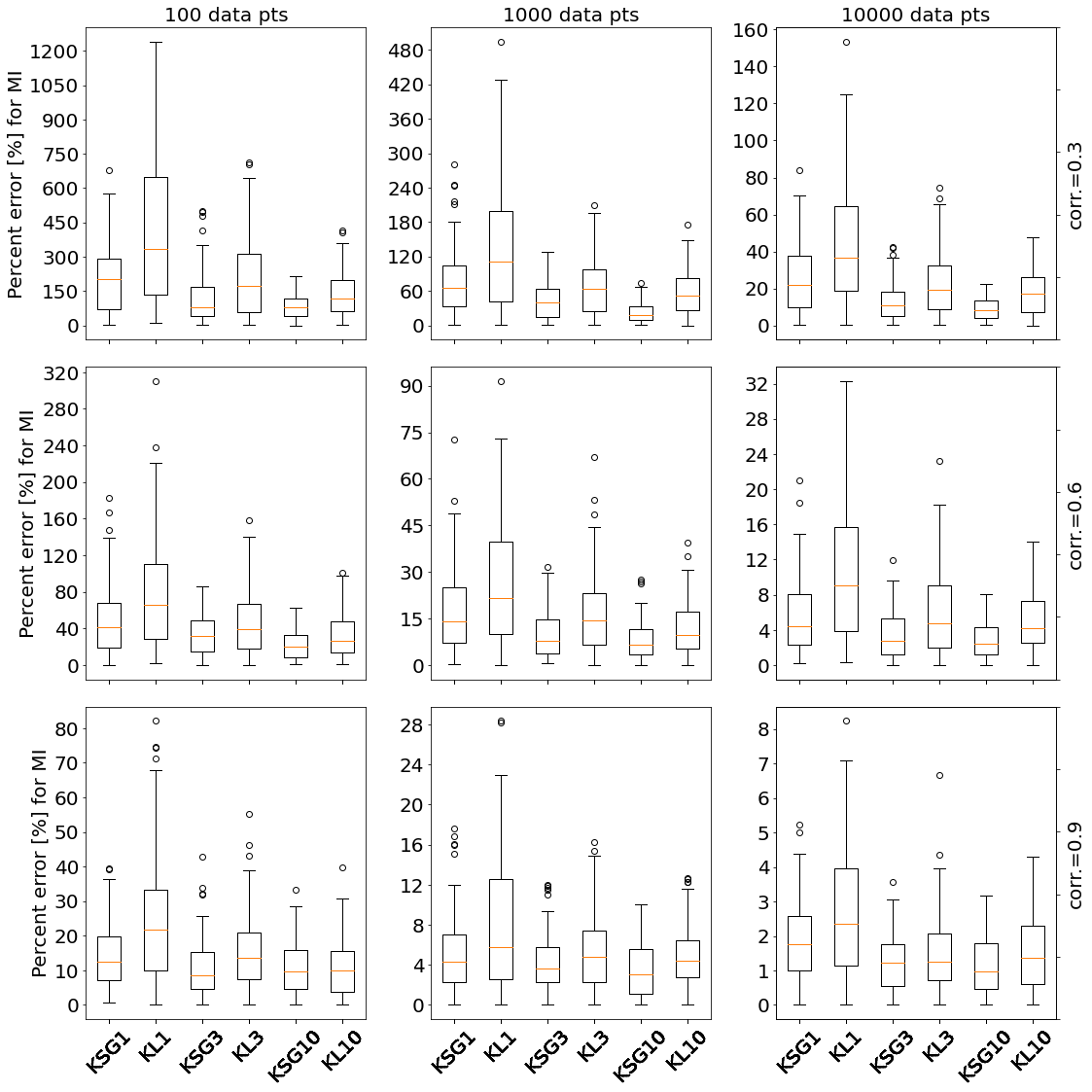
**

**Figure S6**: boxplots of percent error of Total Correlation calculated based on kNN methods for 100 replicates of tri-variate gaussian dist. With sample size = {100,1K,10K}, correlation = {0.3,0.6,0.9}. We compare KL and KSG methods for k=1,3,10. **
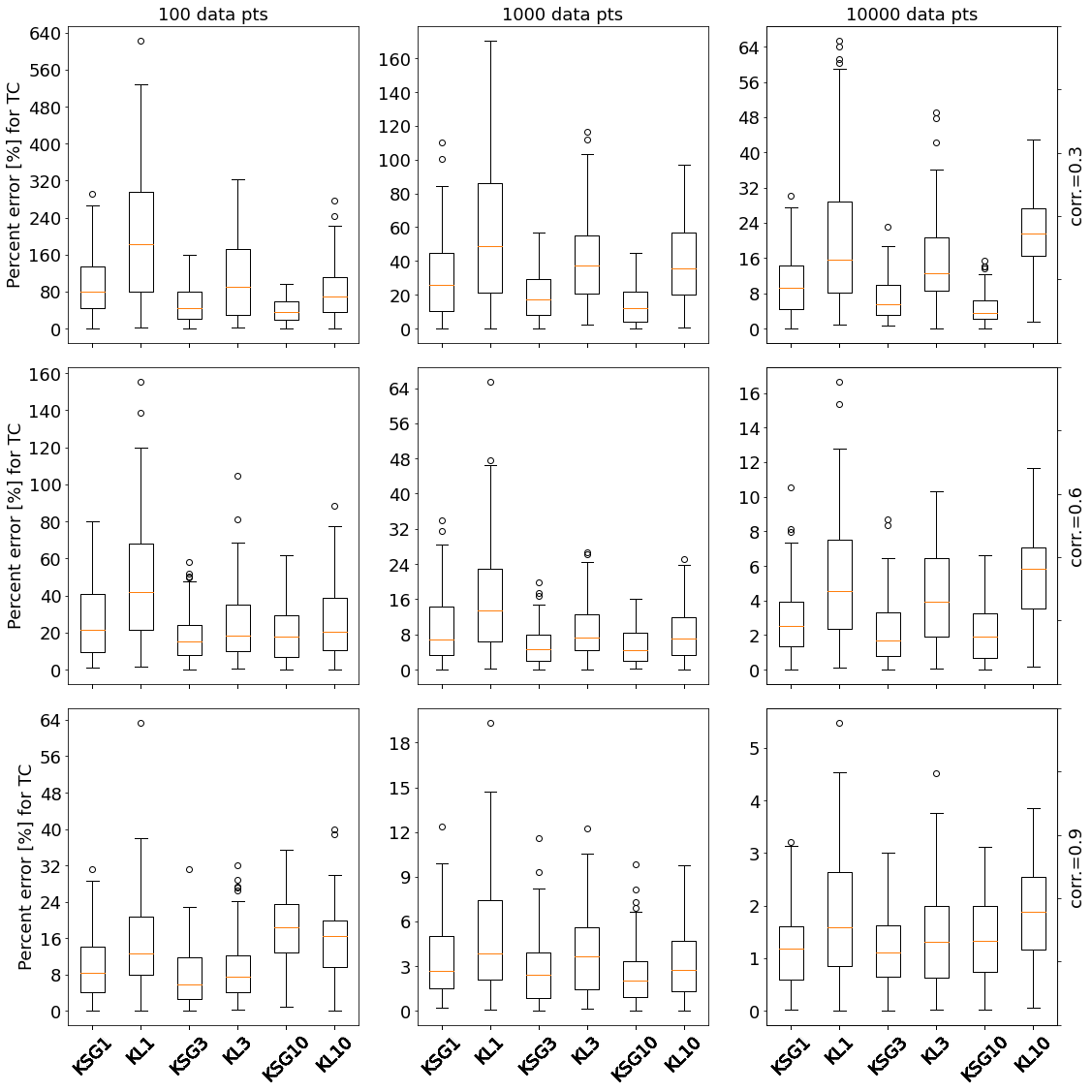
**


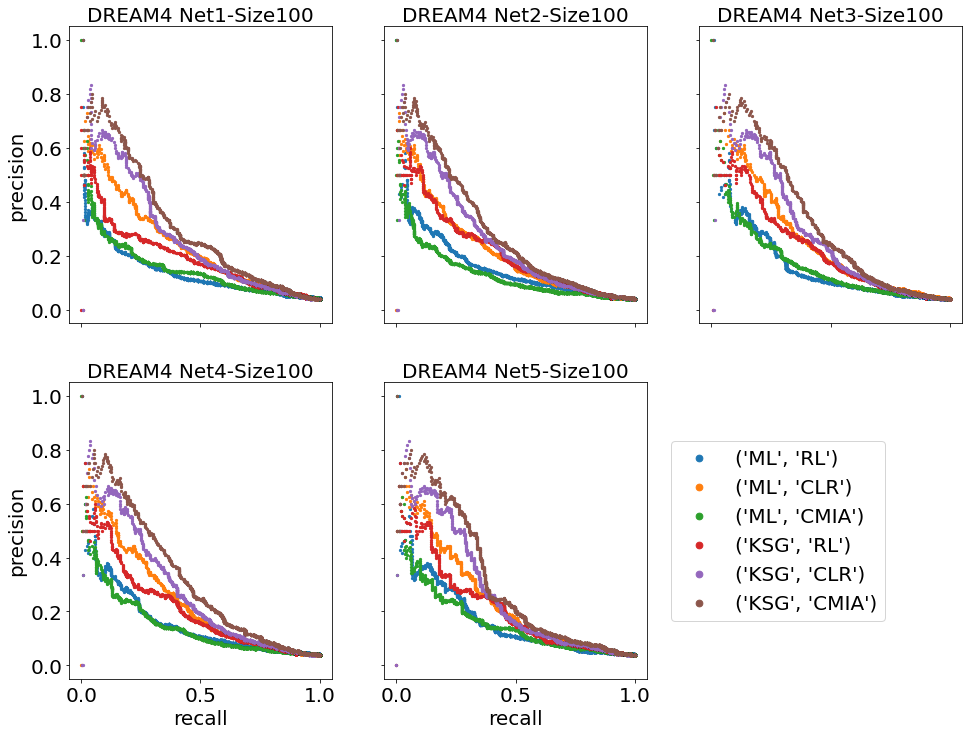
**Figure S7**: Precision-recall curves of six MI-Inference algorithms of five different synthetic networks from DREAM4.

**Figure S8:** Area Under Precision-Recall curve (AUPR) vs. different number of bins or k-neighbors. For the five 50 gene networks from DREAM3, with 10 replicates each, we calculated the AUPR for two inference algorithm and two MI estimator {ML,CLR} and {KSG,CLR} with blue and green dots respectively, and {ML,CMIA} and {KSG,CMIA} with grey and purple dots respectively, for different number of bins for ML, and different number of k-neighbors for KSG. The black dashed vertical line represents k=3 and the solid black line represents #bins = floor(sqrt(data_pts)).


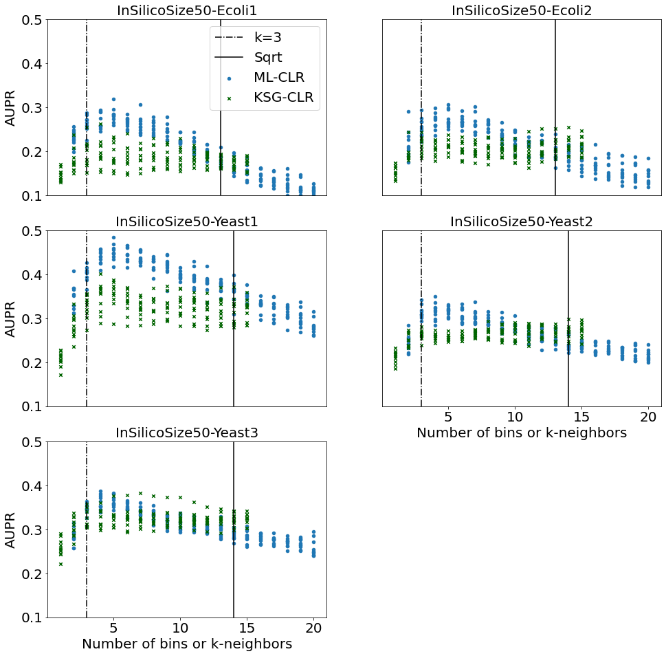

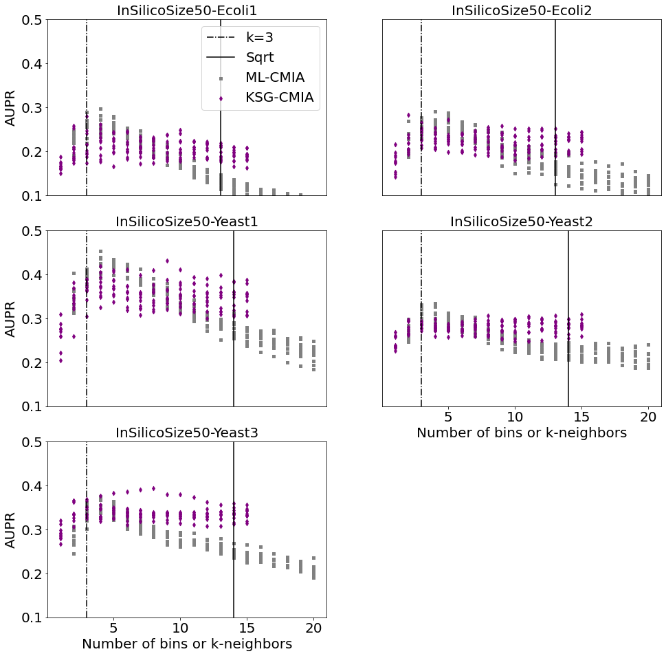


**
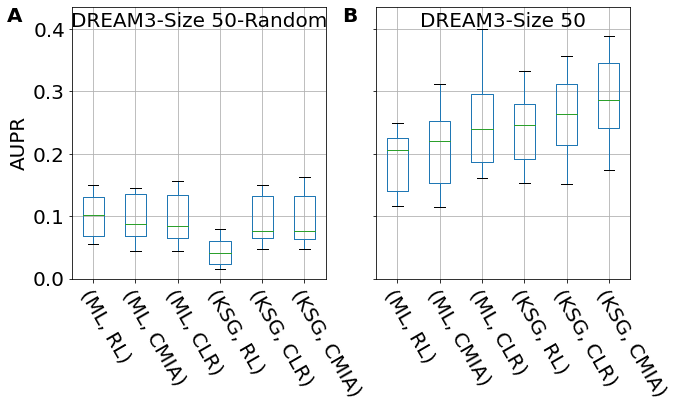
Figure S9:** AUPR for randomized data v.s. true data. Inference of randomized network (A) show significantly lower AUPR than that of the true network (B) across all combinations of MI estimators and inferences algorithms.

**Figure S10:** Comparison of different combinations of MI estimators and inference algorithms used in this work with PIDC and Grnboost2 for networks of different sizes and types. PIDC is based on the ML (maximum likelihood) MI estimator, whereas Grnboost2 does not use an MI estimator.

**
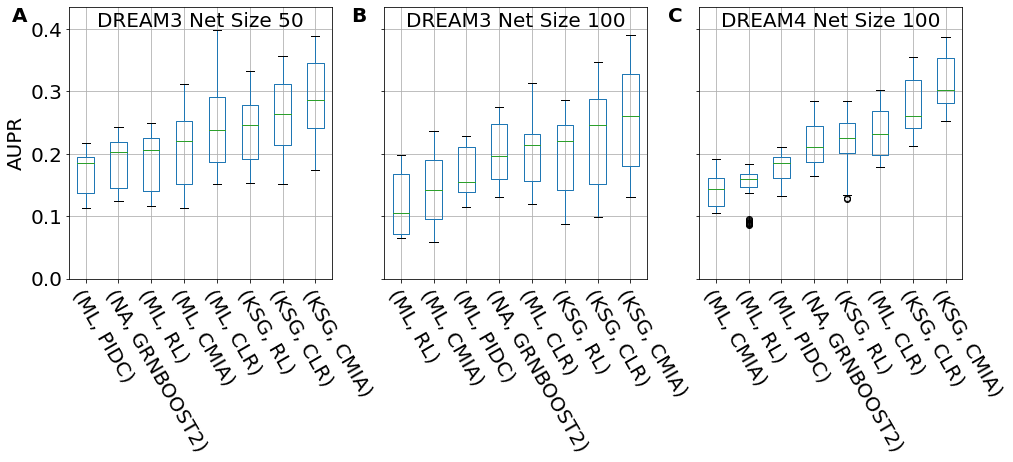
**

**Figure S11**: Common 3-node network motifs

| **No interaction** | **One edge (Two-genes)** | **Fan-out** | **Fan-in** | **Cascade** | **Feed-Forward-Loop (FFL)** |
| --- | --- | --- | --- | --- | --- |
| 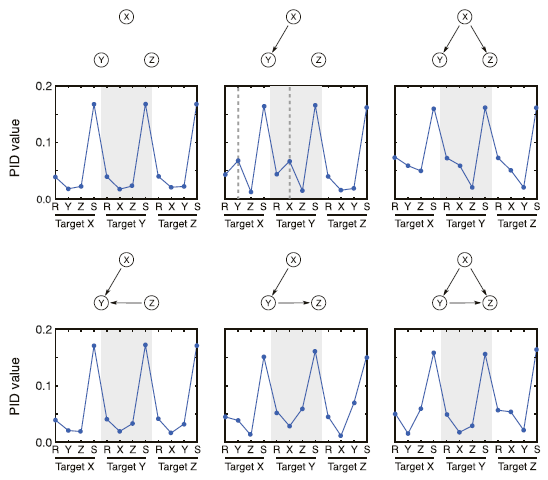 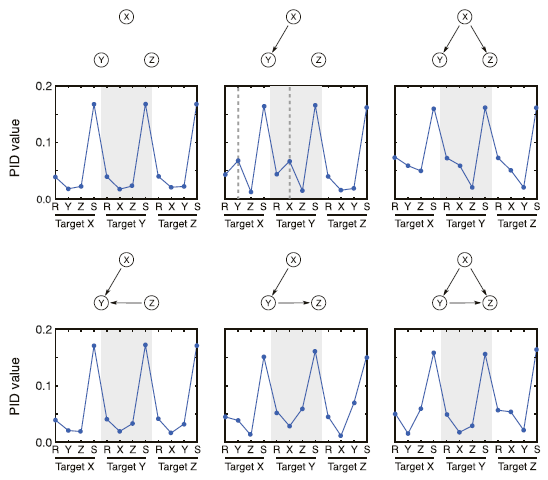 | | | | | |

**Figure S1*2*:** AUPR Performance comparison of GRN reconstruction for different in silico networks modeled from E. coli & Yeast. x-axis shows different combinations of [MI estimator, inference algo], y-axis shows AUPR. (A): Sorted boxplots of the combined four E.coli networks from DREAM3. Each boxplot represents 40 networks (4 different networks X 10 replicates). (B) same as (A) but for the six Yeast networks. (C)-(F): Sorted boxplots of the 4 different E.coli networks from DREAM3. Each boxplot represents 10 replicates. A complete list of tested MI estimators & GRN inference algo can be found in Additional file 2 Table S2


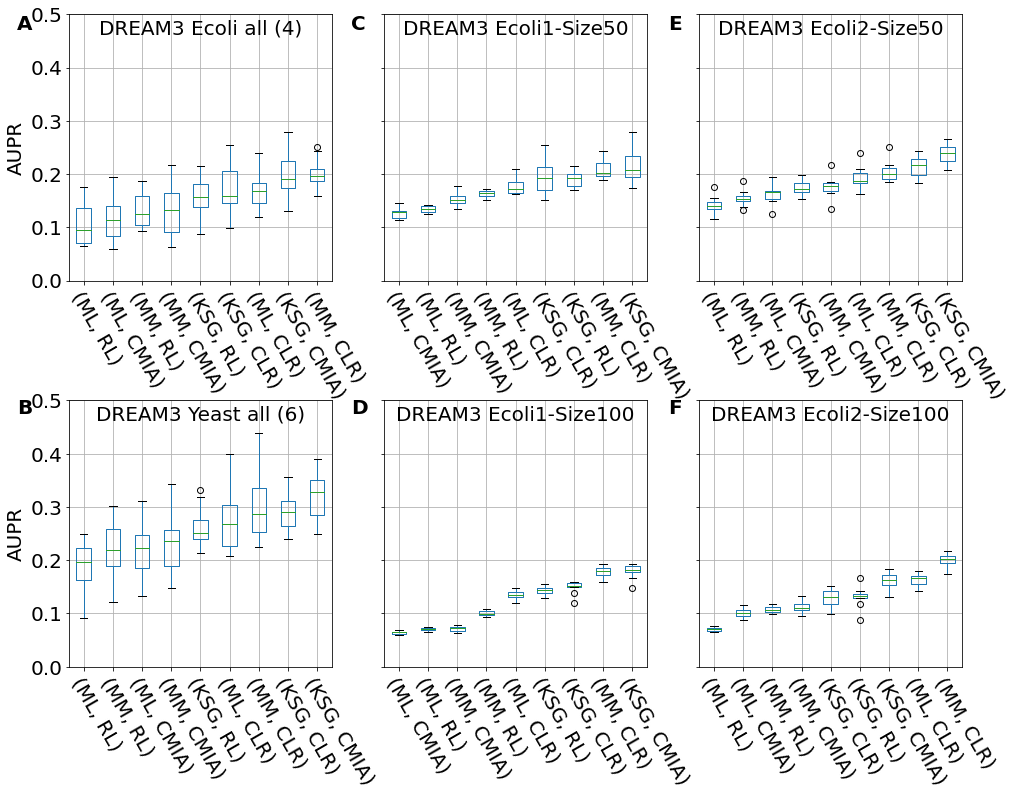


**Figure S13:** *Sorted boxplots of* percentage AUPR difference (increase or decrease) relative to the gold standard combination [ML,CLR] *for different combinations of MI estimator and GRN inference algorithm for the 6 different Yeast networks from DREAM3. Each boxplot represents 10 replicates.*

**
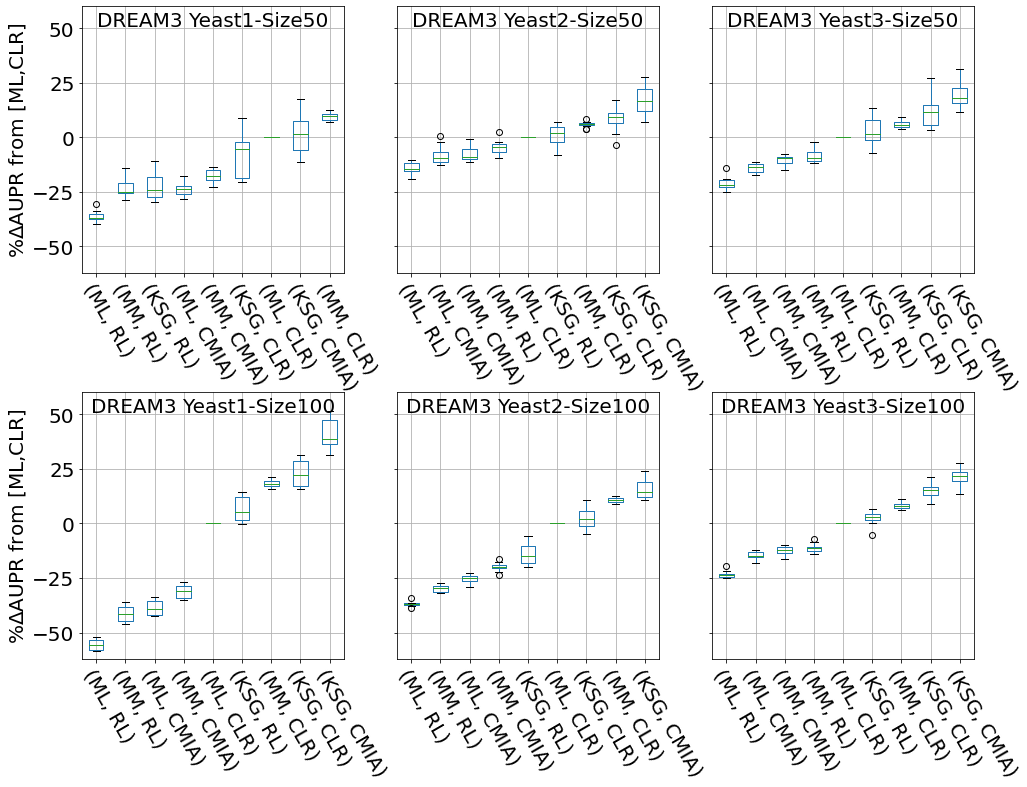
**

**Figure S14:** *Sorted boxplots of* percentage AUPR difference (increase or decrease) relative to the gold standard combination [ML,CLR] *for different combinations of MI estimator and GRN inference algorithm for the 5 different networks of 100 genes from DREAM4. Each boxplot represents 10 replicates.*

**
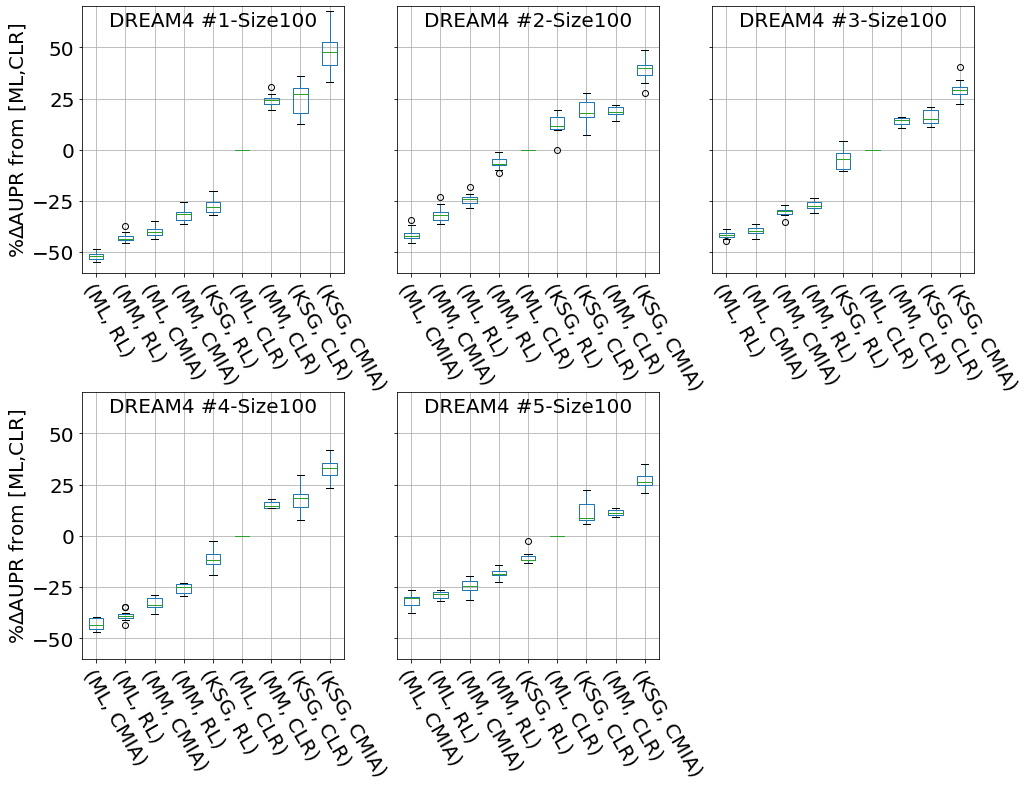
**

**Figure S15:** Area Under Precision-Recall curve (AUPR) vs. different number of bins or k-neighbors, for real *E. coli* data. We calculated the AUPR for two inference algorithm and two MI estimator {ML,CLR} and {KSG,CLR} with blue and green dots respectively, and {ML,CMIA} and {KSG,CMIA} with grey and purple dots respectively, for different number of bins for ML, and different number of k-neighbors for KSG. The black dashed vertical line represents k=3 and the solid black line represents #bins = floor(sqrt(data_pts)).

**
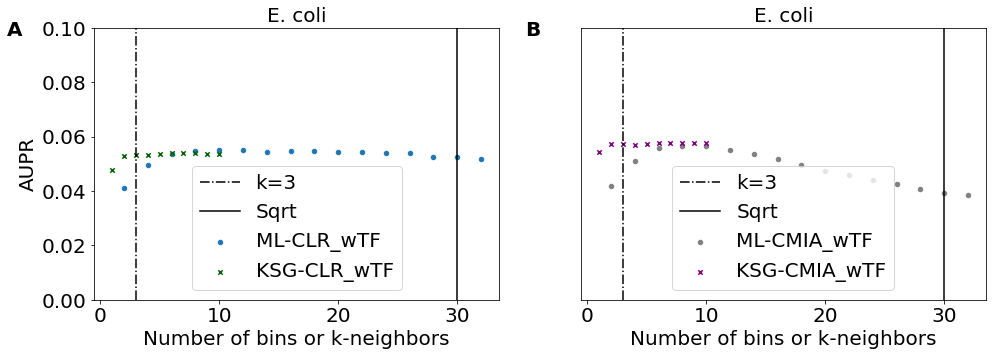
**


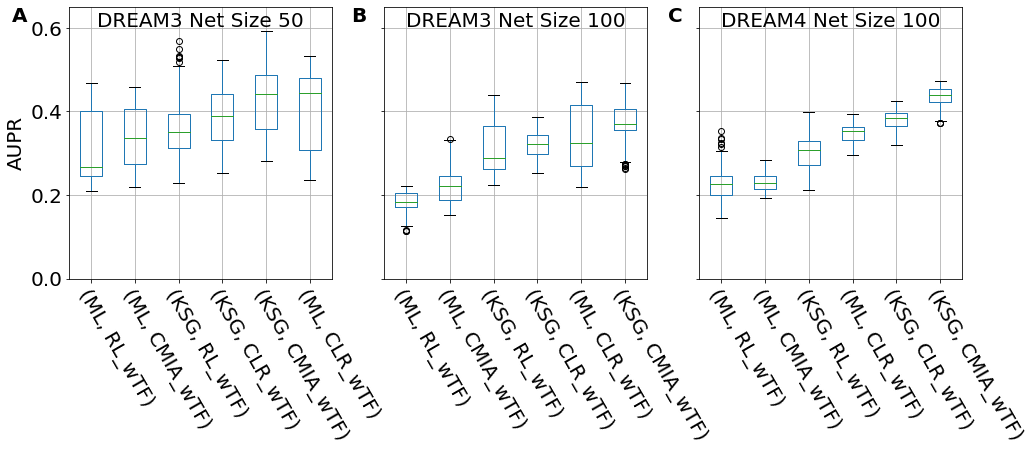
**Figure S16**: AUPR comparison of different combinations of MI estimators and inference algorithms when non-TF-containing interactions are removed from the networks. (A): Sorted boxplots showing networks of size 50 from DREAM3, (B): Networks of size 100 from DREAM3, (C): Networks of size 100 from DREAM4. For the different network sizes each boxplot represents 50 networks (5 different networks X 10 replicates).
