# Supplementary material for: Gene regulation network inference using k-nearest neighbor-based mutual information estimation: revisiting an old DREAM
Source: BMC Bioinformatics. 2023 Mar 6;24:84. doi: 10.1186/s12859-022-05047-5 (PMC9990267; doi:10.1186/s12859-022-05047-5)
Supplement: Supplementary file 3 — Additional file 3. Table S1: Median AUPR and %ΔAUPR (AUPR_relative) values for different combinations of MI estimator and GRN inference algorithm for different network sizes. Table S2: Median AUPR and %ΔAUPR (AUPR_relative) values for different combinations of MI estimator and GRN inference algorithm for different organisms. Table S3: Characteristics of the 10 synthetic networks from DREAM3 and statistics of the different 3-node network motifs extracted. Table S4: precision and recall for various Z-score values for the real E. coli network. [file 12859_2022_5047_MOESM3_ESM.docx]

**Additional file 3: Supplementary information table S1-4**

**Table S1**: Median AUPR and %ΔAUPR (AUPR_relative) values for different combinations of MI estimator and GRN inference algorithm for different network sizes.

| Data set | Network Size | Inf-algo | MI-est | median_AUPR | AUPR_relative |
| --- | --- | --- | --- | --- | --- |
| DREAM3 | 50 | ARACNE | KL | 0.013 | -95.7 |
| DREAM3 | 50 | ARACNE | KSG | 0.136 | -46.6 |
| DREAM3 | 50 | ARACNE | ML | 0.068 | -72.9 |
| DREAM3 | 50 | ARACNE | MM | 0.088 | -64 |
| DREAM3 | 50 | CLR | KL | 0.092 | -64.4 |
| DREAM3 | 50 | CLR | KSG | 0.264 | 7.7 |
| DREAM3 | 50 | CLR | ML | 0.239 | 0 |
| DREAM3 | 50 | CLR | MM | 0.253 | 7.4 |
| DREAM3 | 50 | CMI2rt | KL | 0.008 | -97.1 |
| DREAM3 | 50 | CMI2rt | KSG | 0.068 | -72.6 |
| DREAM3 | 50 | CMI2rt | ML | 0.01 | -96.2 |
| DREAM3 | 50 | CMI2rt | MM | 0.013 | -95 |
| DREAM3 | 50 | CMIA | KL | 0.092 | -62.8 |
| DREAM3 | 50 | CMIA | KSG | 0.285 | 16 |
| DREAM3 | 50 | CMIA | ML | 0.221 | -17.5 |
| DREAM3 | 50 | CMIA | MM | 0.225 | -10.3 |
| DREAM3 | 50 | RL | KL | 0.021 | -93.7 |
| DREAM3 | 50 | RL | KSG | 0.246 | -2 |
| DREAM3 | 50 | RL | ML | 0.206 | -23.5 |
| DREAM3 | 50 | RL | MM | 0.232 | -10.7 |
| DREAM3 | 50 | SA_CLR | KL | 0.084 | -65 |
| DREAM3 | 50 | SA_CLR | KSG | 0.29 | 15.9 |
| DREAM3 | 50 | SA_CLR | ML | 0.188 | -36.8 |
| DREAM3 | 50 | SA_CLR | MM | 0.189 | -33.6 |
| DREAM3 | 100 | ARACNE | KL | 0.018 | -89.4 |
| DREAM3 | 100 | ARACNE | KSG | 0.103 | -50.5 |
| DREAM3 | 100 | ARACNE | ML | 0.051 | -75.2 |
| DREAM3 | 100 | ARACNE | MM | 0.068 | -64.5 |
| DREAM3 | 100 | CLR | KL | 0.062 | -71.6 |
| DREAM3 | 100 | CLR | KSG | 0.246 | 10.4 |
| DREAM3 | 100 | CLR | ML | 0.215 | 0 |
| DREAM3 | 100 | CLR | MM | 0.231 | 17.3 |
| DREAM3 | 100 | CMI2rt | KL | 0.014 | -94 |
| DREAM3 | 100 | CMI2rt | KSG | 0.051 | -76 |
| DREAM3 | 100 | CMI2rt | ML | 0.002 | -99.2 |
| DREAM3 | 100 | CMI2rt | MM | 0.004 | -97.9 |
| DREAM3 | 100 | CMIA | KL | 0.073 | -66.5 |
| DREAM3 | 100 | CMIA | KSG | 0.261 | 22.6 |
| DREAM3 | 100 | CMIA | ML | 0.142 | -35.3 |
| DREAM3 | 100 | CMIA | MM | 0.157 | -28.8 |
| DREAM3 | 100 | RL | KL | 0.03 | -84.6 |
| DREAM3 | 100 | RL | KSG | 0.22 | 0.6 |
| DREAM3 | 100 | RL | ML | 0.105 | -46.8 |
| DREAM3 | 100 | RL | MM | 0.138 | -29.1 |
| DREAM3 | 100 | SA_CLR | KL | 0.07 | -66.2 |
| DREAM3 | 100 | SA_CLR | KSG | 0.259 | 20.1 |
| DREAM3 | 100 | SA_CLR | ML | 0.073 | -57 |
| DREAM3 | 100 | SA_CLR | MM | 0.077 | -56.5 |
| DREAM4 | 100 | ARACNE | KL | 0.002 | -99.1 |
| DREAM4 | 100 | ARACNE | KSG | 0.129 | -43.1 |
| DREAM4 | 100 | ARACNE | ML | 0.08 | -68.3 |
| DREAM4 | 100 | ARACNE | MM | 0.103 | -58.5 |
| DREAM4 | 100 | CLR | KL | 0.03 | -87.9 |
| DREAM4 | 100 | CLR | KSG | 0.26 | 17.8 |
| DREAM4 | 100 | CLR | ML | 0.232 | 0 |
| DREAM4 | 100 | CLR | MM | 0.262 | 15.5 |
| DREAM4 | 100 | CMI2rt | KL | 0.001 | -99.4 |
| DREAM4 | 100 | CMI2rt | KSG | 0.086 | -64.4 |
| DREAM4 | 100 | CMI2rt | ML | 0.001 | -99.5 |
| DREAM4 | 100 | CMI2rt | MM | 0.002 | -99.1 |
| DREAM4 | 100 | CMIA | KL | 0.031 | -87.3 |
| DREAM4 | 100 | CMIA | KSG | 0.302 | 33.6 |
| DREAM4 | 100 | CMIA | ML | 0.144 | -40.1 |
| DREAM4 | 100 | CMIA | MM | 0.169 | -30.5 |
| DREAM4 | 100 | RL | KL | 0.002 | -99 |
| DREAM4 | 100 | RL | KSG | 0.226 | -10 |
| DREAM4 | 100 | RL | ML | 0.159 | -38.7 |
| DREAM4 | 100 | RL | MM | 0.191 | -24 |
| DREAM4 | 100 | SA_CLR | KL | 0.031 | -87.3 |
| DREAM4 | 100 | SA_CLR | KSG | 0.301 | 30.2 |
| DREAM4 | 100 | SA_CLR | ML | 0.076 | -67 |
| DREAM4 | 100 | SA_CLR | MM | 0.082 | -65.2 |

**Table S2**: Median AUPR and %ΔAUPR (AUPR_relative) values for different combinations of MI estimator and GRN inference algorithm for different organisms.

| Organism | Inf-algo | MI-est | median_AUPR | AUPR_relative |
| --- | --- | --- | --- | --- |
| Ecoli | ARACNE | KL | 0.017 | -88.8 |
| Ecoli | ARACNE | KSG | 0.077 | -52.5 |
| Ecoli | ARACNE | ML | 0.033 | -79.8 |
| Ecoli | ARACNE | MM | 0.054 | -66.5 |
| Ecoli | CLR | KL | 0.043 | -70 |
| Ecoli | CLR | KSG | 0.159 | 5.9 |
| Ecoli | CLR | ML | 0.168 | 0 |
| Ecoli | CLR | MM | 0.197 | 19.9 |
| Ecoli | CMI2rt | KL | 0.008 | -94.3 |
| Ecoli | CMI2rt | KSG | 0.032 | -79.6 |
| Ecoli | CMI2rt | ML | 0.003 | -98.6 |
| Ecoli | CMI2rt | MM | 0.003 | -98 |
| Ecoli | CMIA | KL | 0.051 | -66.5 |
| Ecoli | CMIA | KSG | 0.191 | 19.7 |
| Ecoli | CMIA | ML | 0.115 | -33.4 |
| Ecoli | CMIA | MM | 0.133 | -21.9 |
| Ecoli | RL | KL | 0.034 | -76.5 |
| Ecoli | RL | KSG | 0.157 | -2 |
| Ecoli | RL | ML | 0.096 | -38.4 |
| Ecoli | RL | MM | 0.124 | -21.7 |
| Ecoli | SA_CLR | KL | 0.055 | -66.5 |
| Ecoli | SA_CLR | KSG | 0.193 | 19.7 |
| Ecoli | SA_CLR | ML | 0.081 | -53.1 |
| Ecoli | SA_CLR | MM | 0.086 | -51.5 |
| Yeast | ARACNE | KL | 0.016 | -94.7 |
| Yeast | ARACNE | KSG | 0.154 | -46.6 |
| Yeast | ARACNE | ML | 0.077 | -72.2 |
| Yeast | ARACNE | MM | 0.101 | -63.9 |
| Yeast | CLR | KL | 0.096 | -63.6 |
| Yeast | CLR | KSG | 0.291 | 10.5 |
| Yeast | CLR | ML | 0.269 | 0 |
| Yeast | CLR | MM | 0.286 | 8.7 |
| Yeast | CMI2rt | KL | 0.012 | -95.5 |
| Yeast | CMI2rt | KSG | 0.095 | -68.5 |
| Yeast | CMI2rt | ML | 0.01 | -96.2 |
| Yeast | CMI2rt | MM | 0.013 | -95 |
| Yeast | CMIA | KL | 0.101 | -63 |
| Yeast | CMIA | KSG | 0.328 | 18.4 |
| Yeast | CMIA | ML | 0.224 | -18.1 |
| Yeast | CMIA | MM | 0.236 | -14.5 |
| Yeast | RL | KL | 0.024 | -92.4 |
| Yeast | RL | KSG | 0.252 | 0 |
| Yeast | RL | ML | 0.196 | -27.8 |
| Yeast | RL | MM | 0.219 | -14.1 |
| Yeast | SA_CLR | KL | 0.098 | -64.1 |
| Yeast | SA_CLR | KSG | 0.323 | 18 |
| Yeast | SA_CLR | ML | 0.174 | -37.9 |
| Yeast | SA_CLR | MM | 0.177 | -36.5 |

**Table S3**: Characteristics of the 10 synthetic networks from DREAM3 and statistics of the different 3-node network motifs extracted.

| **Network** | **SS data** | **Edges** | **Triplets** | **No Interaction** | **Two-genes** | **Fan-in** | **Cascade** | **Fan-out** | **FFL** | **Sum of 2 edges** | **Sum of 2&3 edges** |
| --- | --- | --- | --- | --- | --- | --- | --- | --- | --- | --- | --- |
| **InSilicoSize100-Ecoli1** | 341 | 125 | 161700 | 150051 | 11059 | 47 | 55 | 477 | 11 | 579 | 590 |
| **InSilicoSize100-Ecoli2** | 322 | 119 | 161700 | 150759 | 10228 | 24 | 51 | 630 | 8 | 705 | 713 |
| **InSilicoSize100-Yeast1** | 401 | 166 | 161700 | 146042 | 15113 | 75 | 212 | 193 | 65 | 480 | 545 |
| **InSilicoSize100-Yeast2** | 401 | 389 | 161700 | 127499 | 30631 | 627 | 1231 | 1361 | 351 | 3219 | 3570 |
| **InSilicoSize100-Yeast3** | 401 | 551 | 161700 | 115759 | 39003 | 1385 | 2052 | 2382 | 1119 | 5819 | 6938 |
| **InSilicoSize50-Ecoli1** | 170 | 62 | 19600 | 16936 | 2361 | 21 | 41 | 232 | 9 | 294 | 303 |
| **InSilicoSize50-Ecoli2** | 169 | 82 | 19600 | 16230 | 2816 | 47 | 20 | 475 | 12 | 542 | 554 |
| **InSilicoSize50-Yeast1** | 201 | 77 | 19600 | 16204 | 3126 | 43 | 103 | 94 | 30 | 240 | 270 |
| **InSilicoSize50-Yeast2** | 201 | 160 | 19600 | 13056 | 5536 | 241 | 306 | 333 | 128 | 880 | 1008 |
| **InSilicoSize50-Yeast3** | 201 | 173 | 19600 | 12629 | 5812 | 195 | 303 | 487 | 174 | 985 | 1159 |

**Table S4**: precision and recall for various Z-score values for the real E. coli network. Where TP=True positives, FP=False positives, FN=False negative.

| **Index** | **Edge** | **Z-score** | **TP** | **TPsum** | **FP** | **FPsum** | **FNsum** | **precision** | **recall** |
| --- | --- | --- | --- | --- | --- | --- | --- | --- | --- |
| 0 | G742-G743 | 20.0688 | 1 | 1 | 0 | 0 | 3469 | 1 | 0.0003 |
| 1 | G137-G138 | 19.6224 | 1 | 2 | 0 | 0 | 3468 | 1 | 0.0006 |
| 3 | G855-G1518 | 18.4701 | 1 | 4 | 0 | 0 | 3466 | 1 | 0.0012 |
| 5 | G1095-G1098 | 17.3448 | 1 | 6 | 0 | 0 | 3464 | 1 | 0.0017 |
| 8 | G497-G498 | 16.2632 | 1 | 9 | 0 | 0 | 3461 | 1 | 0.0026 |
| 11 | G327-G417 | 15.3402 | 1 | 12 | 0 | 0 | 3458 | 1 | 0.0035 |
| 14 | G704-G705 | 14.1502 | 1 | 14 | 0 | 1 | 3456 | 0.9333 | 0.004 |
| 22 | G585-G1240 | 13.0574 | 1 | 21 | 0 | 2 | 3449 | 0.913 | 0.0061 |
| 31 | G585-G1241 | 12.1214 | 1 | 29 | 0 | 3 | 3441 | 0.9062 | 0.0084 |
| 41 | G21-G350 | 11.1091 | 0 | 35 | 1 | 7 | 3435 | 0.8333 | 0.0101 |
| 50 | G180-G185 | 10.0211 | 1 | 43 | 0 | 8 | 3427 | 0.8431 | 0.0124 |
| 72 | G292-G1343 | 9.0216 | 0 | 51 | 1 | 22 | 3419 | 0.6986 | 0.0147 |
| 128 | G631-G786 | 8.0253 | 0 | 72 | 1 | 57 | 3398 | 0.5581 | 0.0207 |
| 208 | G142-G1541 | 7.0028 | 0 | 103 | 1 | 106 | 3367 | 0.4928 | 0.0297 |
| 365 | G1001-G1598 | 6.0009 | 0 | 128 | 1 | 238 | 3342 | 0.3497 | 0.0369 |
| 856 | G49-G877 | 5.0008 | 0 | 189 | 1 | 668 | 3281 | 0.2205 | 0.0545 |
| 2170 | G743-G1553 | 4.0004 | 0 | 271 | 1 | 1900 | 3199 | 0.1248 | 0.0781 |
| 7121 | G72-G632 | 3.0001 | 0 | 467 | 1 | 6655 | 3003 | 0.0656 | 0.1346 |
